# The projack: a resampling approach to correct for ranking bias in high-throughput studies

**DOI:** 10.1093/biostatistics/kxv022

**Published:** 2015-06-03

**Authors:** Yi-Hui Zhou, Fred A. Wright

**Affiliations:** Department of Biological Sciences, Bioinformatics Research Center, North Carolina State University, NC 27695, USA; Departments of Statistics and Biological Sciences, Bioinformatics Research Center, North Carolina State University, NC 27695, USA

**Keywords:** Bias correction, Effect size estimation, Winner's curse

## Abstract

The problem of ranked inference arises in a number of settings, for which the investigator wishes to perform parameter inference after ordering a set of $m$ statistics. In contrast to inference for a single hypothesis, the ranking procedure introduces considerable bias, a problem known as the “winner's curse” in genetic association. We introduce the *projack* (for Prediction by Re- Ordered Jackknife and Cross-Validation, $K$-fold). The projack is a resampling-based procedure that provides low-bias estimates of the expected ranked effect size parameter for a set of possibly correlated $z$ statistics. The approach is flexible, and has wide applicability to high-dimensional datasets, including those arising from genomics platforms. Initially, motivated for the setting where original data are available for resampling, the projack can be extended to the situation where only the vector of $z$ values is available. We illustrate the projack for correction of the winner's curse in genetic association, although it can be used much more generally.

## 1. Introduction

We consider the classical setting in which a vector of statistics ${\textbf{z}}=\{z_1, z_2, \ldots , z_m\}$ is observed, where $Z_i \sim N(\mu _i,1),\ i=1, \ldots , m,$ and $\boldsymbol \mu =\{\mu _1, \mu _2,\ldots ,\mu _m\}$ is fixed and unknown. We refer to each $i$ as corresponding to a “hypothesis,” although our focus is mainly on estimation rather than testing. A vast literature, mostly assuming independence of the statistics, concerns estimation of $\boldsymbol \mu$ under a squared error loss. Key approaches and seminal work includes parametric and nonparametric empirical Bayes (Robbins, 1956), James–Stein shrinkage (James and Stein, 1961, Efron and Morris, 1973) and, for “sparse” $\boldsymbol \mu$ consisting of many zeros, testing-based threshold approaches (e.g., Johnstone and Silverman, 2004), with approaches concisely reviewed in Jiang and Zhang, 2009).

However, in large-scale multiple testing problems, the researcher is primarily interested in performing parameter inference for those hypotheses showing the greatest evidence against the null hypothesis, often after performing significance testing corrected for multiple comparisons. Any inference concerning the significant hypotheses must account for the impact of the selection procedure itself, for otherwise the estimates will be hopelessly biased (Ghosh *and others*, 2008). In genomics, this selection bias, termed the “winner's curse,” can be extreme, as the significance testing thresholds must account for thousands or even millions of tests.

### 1.1. The ranked inference problem

Without loss of generality, we assume that subscripts have been assigned so that $\boldsymbol \mu$ is sorted, i.e., each $\mu _i \le \mu _{i+1}$, although $\boldsymbol \mu$ and the true ordering are unknown to the data analyst. We use “$(i)$” to denote the original index of the $i$th ranked $z$ value. The order statistics are ${\textbf{z}}_{(.)}=\{z_{(1)}, z_{(2)},\ldots ,z_{(m)}\}$, with correspondingly ordered parameters ${{\boldsymbol \mu }_{(.)}}=\{\mu _{(1)},\ldots ,\mu _{(m)}\}$. The indexes $(.)=\{(1), (2), \ldots , (m)\}$ are determined by the random outcome ${\textbf{z}}$, and so ${\boldsymbol \mu }_{(.)}$ is random, even though $\boldsymbol {\mu }$ is fixed.

The broad goal of our ranked inference is to find, for each $i$, an estimate of $\delta _{(i)}=E(\mu _{(i)})$ with low bias and error, where the expectation is over realizations of ${\textbf{z}}$. A fully successful procedure should work for any $\boldsymbol \mu$ and all $i$, although hypotheses with extreme $z$ values are considered most important.

### 1.2. An example from a psoriasis genome-wide association study

Table 1 shows odds ratios for allele effects of 10 SNP markers reported in Nair *and others* (2009) in a case–control study of 1350 psoriasis cases vs. 1400 controls. The study is illustrative because the authors report odds ratios from a replication set of 5000 cases and 5000 controls (larger than the initial scan), and these data were later analyzed by Faye *and others* (2011) using a proposed winner's curse correction method. The SNPs were among the most significant among 480 000 SNPs tested in the initial study. We treat the replication odds ratios as a gold standard, because they are not subject to the winner's curse, and are based on a larger sample size. Here we see direct evidence of the winner's curse, in which all of the replication odds ratios are closer to 1 than the original estimates, and we show below how to relate the odds ratios to $\boldsymbol \mu$. The bias in the naive initial estimates can have several negative consequences, including (i) exaggerated attributable risk calculations for the SNPs, (ii) overly optimistic sample size calculations in planning replication studies, and (iii) the possibility of falsely declaring an SNP as “not replicated” in a follow-up study, based on unrealistically high estimation of the true effect size. Interestingly, the SNP with the highest initial odds ratio ($\mbox {OR}=2.78$ for rs12191877 near *HLA-B*) shows comparatively little shrinkage under replication, due to the phenomenon that the winner's curse has little effect on hypotheses with extreme test statistics (Ghosh *and others*, 2008).

**Table 1.** Ten SNPs displaying association with psoriasis in Nair *and others* (2009). Genotypes were coded so that odds ratios for these SNPs are $>$1

Chr	SNP	Pos(Mb)	Nearest gene	z	$p$-value	Naive OR	Repl. OR
6	rs12191877	31.36	*HLA-B*	15.34	$3.9 \times 10^{-53}$	2.79	2.64
5	rs2082412	158.65	*UBLCP1*	6.20	$5.5 \times 10^{-10}$	1.56	1.44
5	rs17728338	150.46	*ANXA6*	5.20	$2.0\times 10^{-7}$	1.72	1.59
5	rs20541	132.02	*IL13*	4.53	$5.8\times 10^{-6}$	1.37	1.27
6	rs610604	138.24	*TNFAIP3*	4.34	$1.4\times 10^{-5}$	1.28	1.19
12	rs2066807	55.02	*STAT2*	4.33	$1.5\times 10^{-5}$	1.68	1.34
1	rs2201841	67.47	*IL23R*	5.13	$2.9\times 10^{-7}$	1.35	1.13
9	rs1076160	134.8	*TSC1*	4.24	$2.2\times 10^{-5}$	1.26	1.09
19	rs12983316	10.98	*SMARCA4*	4.23	$2.3\times 10^{-5}$	1.37	1.09
2	rs397211	113.6	*IL1RN*	3.29	$1.00\times 10^{-3}$	1.21	1.08

We note that replication results for a few markers are not always available for genome-scan data. Moreover, replication of an entire GWAS on the same platform gives rise to new combined results ($z$-statistics) on the initial and replication data, and the combined analysis is again subject to the winner's curse. With the advent of large meta-analyses and “mega”-analyses across numerous cohorts, the results are inherently high dimensional, and often without replication of a few significant markers. Thus it is crucial that the best estimates of effect size be extracted from the same data used to rank the genomic features. Following the example from Table 1, we seek a procedure that can take the initial data and predict the “true” parameter values (odds ratios, in our example), without requiring replication.

### 1.3. Existing approaches for the ranked inference problem

For *a priori* fixed significance testing thresholds, using a likelihood conditional on significance (Ghosh *and others*, 2008; Zhong and Prentice, 2008) offers a principled approach to correct the winner's curse, provided a fixed significance threshold $c$ (i.e., reject when $|z|>c$) is used. For $Z\sim N(\mu ,1)$ and unknown $\mu$, the conditional likelihood is

\[L_c(\mu )=p_\mu (z |\, |Z| >c)=\frac {p_\mu (z)}{P_\mu (|Z|>c)}=\frac {\phi (z-\mu )}{\Phi (-c+ \mu )+ \Phi (-c-\mu )},\]

where $\Phi$ is the standard normal cdf, and we might use $\tilde \mu ={\rm argmax}_\mu L_c(\mu )$ as an estimate of the corresponding $\delta$. However, the dependence on the threshold is not entirely satisfying. If $c$ is chosen based on a genome-wide threshold (i.e., over all tested hypotheses), this approach would not even apply to most of the SNPs in Table 1, as they did not meet multiple testing criteria (e.g., the Bonferroni threshold for the initial study would be $\sim 1\times 10^{-7}$ for family-wise error $\alpha =0.05$).

Here, as a competitor to our proposed procedure, we use a slight modification to the conditional likelihood approach, as described in Ghosh *and others* (2014). The modified conditional likelihood uses $\hat {\delta }=\tilde \mu$ if $|z|>c$ and 0 otherwise, and is performed for several values of $c$. In order to capture a larger number of hypotheses, $c$ might be chosen to correspond to nominal significance, uncorrected for genome-wide multiple comparisons. Larger values of $c$, e.g., corresponding to genome-wide significance, will aggressively shrink all but the most extreme statistics.

If the full original data are available, then resampling approaches are an alternative. Faye *and others* (2011) proposed a bootstrapping association approach implemented as the *BR-Squared* software, designed for genetic association and with individuals as the sampled units. The non-sampled individuals (“out-of-bag”, $\sim$37% of the data) are used as an artificial replication sample for parameter estimation. However, this approach creates new difficulties, because resampling from the fixed dataset requires handling complex correlations between the in-sample and out-of-sample parameter estimates (Faye *and others*, 2011). In addition, the approach requires a full analysis of each resampled dataset, which can be computationally demanding. Moreover, the software cannot handle “dosage” genotypes (Zhang *and others*, 2011) which result from imputation, which are increasingly commonly used.

In this paper, we propose an approach that essentially works with split data samples, using a training portion of the data to rank hypotheses, and the remaining (test) portion to predict effect sizes for the ranked hypotheses. Similar procedures have been proposed (Sun and Bull, 2005), but our procedure has several novel advantages, including (i) a pseudo-matrix construction that generalizes, simplifies, and speeds up resampling; (ii) a procedure to flexibly handle bias/variance tradeoffs in estimation; and (iii) an extension to handle the common situation that only the vector ${\textbf{z}}$ is available.

### 1.4. Connecting ${\mu }$ to standard parameterization

The analysis of a vector ${\textbf{z}}$ arises in a huge variety of applications, as diverse as the comparison of toxoplasmosis infection rates (Efron and Morris, 1975) and genetic association studies (Ghosh *and others*, 2008), etc. Typically, we model the original data for feature $i$ as governed by a parameter of interest $\beta _i$ with standard error ${{\rm SE}}_i$. A Wald or similar statistic is computed as $z_i={\hat \beta }_i/{\widehat {\rm SE}_i}$, where “$\hat {\ }$” signifies an estimate, and $\mu _i =\beta _i/{\rm SE}_i$. In the example from Table 1, $\beta$ is the natural log of the odds ratios for the effect of each SNP allele. Our basic approach is to use ${\textbf{z}}$ to perform inference for $\boldsymbol \mu$, and then convert back to the $\beta$ scale by multiplying by the original estimated standard errors (e.g., as in the toxoplasmosis data of Efron and Morris, 1975 or genetic association data of Ghosh *and others*, 2008). Technically, the fact that standard errors are estimated produces a slight additional bias, such that $E(Z_i)\ne \mu _i$, even if $E(\hat {\beta }_i)=\beta _i$. However, this bias is not consequential to the treatment here, as demonstrated in numerous simulations and analyses.

## 2. The projack method

The basic projack has several key elements: (i) construction of an $m\times n$ pseudo-matrix to serve as the data object for resampling; (ii) a re-ordered jackknife procedure to produce low-bias estimates of $\boldsymbol \delta$; and (iii) $K$-fold stabilization to ensure that the variance of the estimates is not too large. We will use $m \times n$ matrix ${\textbf{C}}$ as a notational example. The $i, j$ element is $c_{ij}$, the $i$th row vector ${\textbf{c}}_{i.}$, and $j$th column vector ${\textbf{c}}_{.j}$. Where necessary, a random matrix/vector will be denoted using boldface italics (e.g., ${\boldsymbol{{ {C}}}},{\boldsymbol{{ {c}}}}$), and scalar random variables by capital italic (e.g., $C_{ij}$). We use $r({\textbf{C}})$ to denote the $m\times 1$ vector of row means of ${\textbf{C}}$. We use a bar to represent the mean of a vector e.g., $\bar {{\textbf{c}}}_{i.}$. ${\textbf{C}}_{(.)}$ denotes the result of re-ordering rows of ${\textbf{C}}$ so that its row means are increasing. The addition or subtraction of a vector to a matrix is column-wise, so that, for example, ${{\textbf{C}}}-r({\textbf{C}})$ is an $m \times n$ matrix with elements $c_{ij}-\sum _{j^\prime }c_{i j^\prime }/n$. Finally, we use subscript $[-j]$ to signify removal of the $j$th column.

### 2.1. Construction of a pseudo-matrix from original data

For many problems, the original data can be divided into an $m \times n$ matrix ${\textbf{X}}$ and length-$n$ vector ${\textbf{y}}$, with perhaps an additional $p \times n$ covariate matrix ${\textbf{V}}$. The statistics result from a comparison of each row ${\textbf{x}}_{i.}$ to ${\textbf{y}}$ and ${\textbf{V}}$, i.e., $z_i=\hat {\mu }_i=f({\textbf{x}}_{i.}, {\textbf{y}},{\textbf{V}})$. In a genomics context, ${\textbf{X}}$ might be genotype or gene expression data, with ${\textbf{y}}$ a vector of clinical outcomes or experimental conditions. The relationship between ${\textbf{X}}$ and ${\textbf{y}}$ is of primary interest, but might be moderated by covariates ${\textbf{V}}$. Here $f$ will typically be a Wald or other $z$-statistic.

**Fig. 1.**
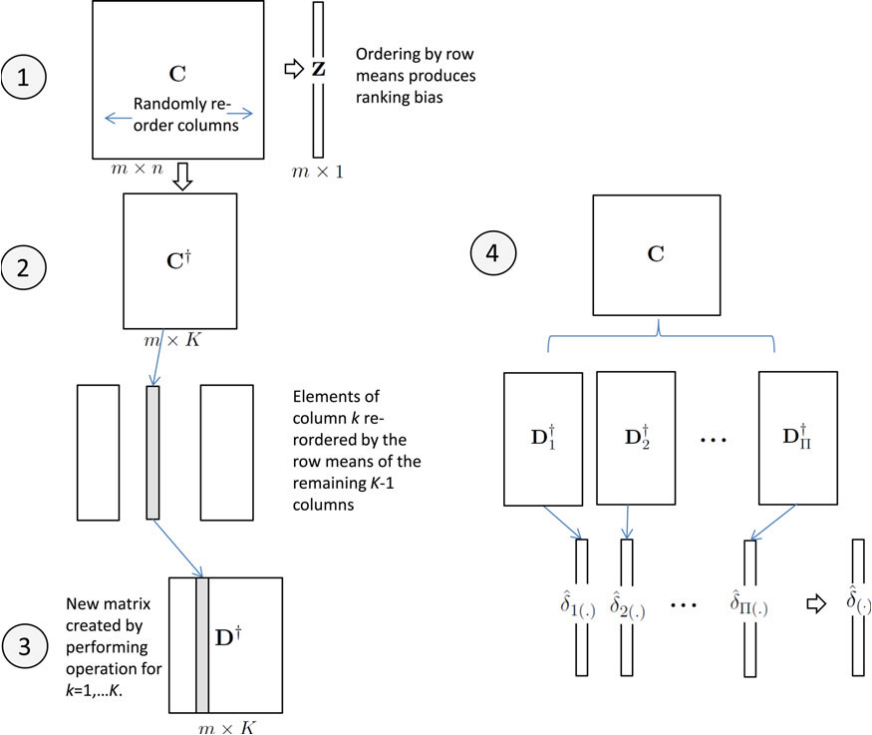
Flowchart of projack-$K$.

The projack approach works by performing split-sample analysis of a single matrix, and we construct an appropriate matrix as follows, using a kind of jackknife pseudo-value. Define the $m \times n$ matrix ${\textbf{C}}$ with elements $c_{ij}=n \hat {\mu _i} - (n-1)\hat {\mu }_{i[-j]}$, where $\hat {\mu }_{i[-j]}=z_{i[-j]}\sqrt {n/(n-1)}$ and $z_{i[-j]}=f({\textbf{x}}_{i[-j]}, {\textbf{y}}_{[-j]},{\textbf{V}}_{[-j]})$. Our construction is designed to ensure that $r({\textbf{C}})\approx {\textbf{z}}$ (Figure 1), so that the resampling approach can work directly with ${\textbf{C}}$ rather than the original data. Appendix A of Supplementary material describes the process in more detail. For complex statistics involving nuisance parameters and GWAS-size data, computing ${\textbf{C}}$ can be computationally intensive. However, for a large number of settings, score statistics can be used, are asymptotically equivalent to Wald and likelihood ratio statistics under the null, and are typically very close to these statistics even under the alternative. We describe in Appendix A of Supplementary material how the score statistic approach can be used to provide a fast and accurate approximation to ${\textbf{C}}$ without requiring the leave-one-out pseudovalue computation.

### 2.2. Prediction via the re-ordered jackknife

Due to ranking bias, $z_{(i)}$ itself is typically a poor estimator of $\delta _{(i)}$, unless the statistic is an isolated extreme value. The column structure of ${\textbf{C}}$ enables a drastic reduction in bias. Consider the new matrix ${\textbf{C}}_{[-j]}$, which is identical to ${\textbf{C}}$, except for removal of the $j$th column. We use $(i)_{[-j]}$ as the index of the $i$th ranked row mean of ${\textbf{C}}_{[-j]}$. If $n$ is large, then ranking based on ${\textbf{C}}_{[-j]}$, which uses all but a single sample, typifies the behavior for sample size $n$. However, because the $j$th column has not been used for ranking, it is not subject to ranking bias. Accordingly, we use the element $c_{(i)_{[-j]},j}$ as indicative of $\mu _{(i)}$ for each $i$.

Using a single held-out column for our prediction is inefficient, so we proceed by performing the re-ordering procedure for each $j=1,\ldots ,n$ to create a new re-ordered matrix ${\textbf{D}}$, where $d_{ij}=c_{(i)_{[-j]},j}$. Finally, we construct the estimator as $r({\textbf{D}})$, i.e., $\hat {\delta }_{(i)}=\sum _{j=1}^n d_{ij}/n$. Note that $\hat {\delta }_{(i)}$ serves as both an estimator of $\delta _{(i)}$ and a predictor of $\mu _{(i)}$. We refer to this leave-one-out procedure, conducted for each $j$, as the *re-ordered jackknife*. We note that for the idealized data structure, and if the effect sizes are constant, i.e., $\mu _i=\mu _0,$ for each $i\in \{1,\ldots ,n\}$, the re-ordered jackknife is exactly unbiased. This follows from the fact that the row means of ${\textbf{C}}_{[-j]}$ are independent of ${\textbf{c}}_{.j}$. It further follows that each element of $d_{ij}$, and therefore $\hat \delta _{(i)}$, has expectation $\mu _0$. Table 1 and Appendix B of Supplementary material illustrate the re-ordering procedure for a small dataset, with $m=5$, $n=4$, obtained by drawing each element as iid $N(0,n)$, i.e., $\mu _0=0$. Most of the $\hat \delta _{(.)}$ values have been shrunken toward zero in comparison with $z_{(.)}$, as expected.

### 2.3. $K$-fold stabilization

The re-ordered jackknife has only slight bias, as each of the re-orderings is based on $n-1$ samples. However, the leave-one-out estimate is highly variable. Alternatively, we might accept somewhat greater bias in order to produce more stable estimates. We propose a cross-validation procedure in which columns are divided into $K$ equal subsets, and each portion held out in succession. We perform the cross-validation for a large number of random $K$-fold partitions of the data, providing the extra stability. For a single partition, we let $J_k$ denote the set of $n/K$ samples belonging to the $k$th subset. Define $c^\dagger _{ik}=(K/n) \sum _{j\in J_k} c_{ij}$ to create a new $m\times K$ matrix ${\textbf{C}}^\dagger$. It is simple to show by central limit arguments that the row mean of ${\textbf{C}}^\dagger _{i.}$ is still approximately $N(\mu _i,1)$, and for rank bias correction we can perform the re-ordered jackknife on ${\textbf{C}}^\dagger$ to create a matrix ${\textbf{D}}^\dagger$. Performing the procedure for each random partition $\pi =1,\ldots ,\Pi$, we produce re-ordered matrices ${\textbf{D}}_1^\dagger ,{\textbf{D}}_2^\dagger , \ldots , {\textbf{D}}_\Pi ^\dagger$, with elements $d^\dagger _{ik\pi }$. Finally, we average over the $\Pi$ random partitions to produce $\hat {\delta }_{(i)}=(\sum _{\pi =1}^{\Pi } \sum _{k=1}^K d^\dagger _{ik\pi })/(\Pi K)$. Together, the re-ordered jackknife and $K$-fold stabilization/cross-validation are a coherent procedure to estimate the average effect size for ranked hypotheses, as shown in Figure 1. We refer to the entire procedure as the *projack*, for Prediction by Re-Ordered Jackknife and Cross-Validation, $K$-fold.

### 2.4. Performance for $K=5$ and $K=10$, and for small samples

We propose a choice of $K$ in the range of 5$-$10 as striking a reasonable balance between bias and variability (similar to other work on cross-validation, Kohavi, 1995). Here we illustrate the $\hat {\boldsymbol \delta }_{(.)}$ bias and coverage performance with $n=50$ samples and $m=100$ hypotheses. We consider a variety of $\boldsymbol \mu$ vectors: (a) the null scenario where all $\mu _i=\mu _0$, and without loss of generality we use $\mu _0=0$; (b) a “sparse” scenario in which all but three hypotheses are null, ${\boldsymbol \mu }=\{-6,-3,0,\ldots ,0,2\}$; (c) $\boldsymbol \mu$ exactly following a standard normal empirical cdf, i.e., $\mu _i = \Phi ^{-1}(i/(m+1))$ for standard normal cdf $\Phi$; (d) the “box”-shaped uniform $\boldsymbol \mu$ where the $\mu _i$ are equally spaced on the interval $[-2,2]$. For each of the scenarios, we performed 1000 simulations, using $\Pi =50$ splits throughout. For each simulated matrix ${\textbf{C}}$, we used two choices of $K$ (5 and 10). In addition, we performed separate simulations in which the rows of ${\textbf{C}}$ were uncorrelated with $\rho =0$, or were moderately correlated with common $\rho =0.4$.

**Fig. 2.**
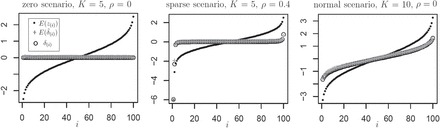
The low bias of the projack is illustrated for several scenarios described in the text.

**Fig. 3.**
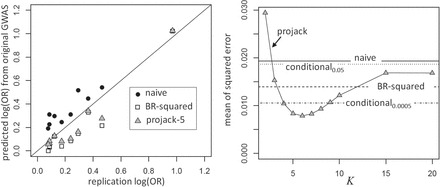
Results for the 10 SNPs from the psoriasis dataset. Left panel: Estimates based on original GWAS ($y$-axis) vs. replication values used as a “gold standard” ($x$-axis). Right panel: Mean of $(\hat {\beta }_i-\beta _i)^2$ values across the SNPs for the various bias-correction approaches.

Figure 2 shows $E(z_{(i)})$, $E(\hat {\delta }_{(i)})$, and the true $\delta _{(i)}$ vs. $i$ for three of the 16 scenarios. The results for all 16 scenarios as well as mean-squared error (MSE) results are shown in Figures 3 and 4 of Supplementary material. Although $E({\boldsymbol{{ {z}}}}_{(.)})$ shows the clear bias as anticipated, both projack-5 and projack-10 are nearly unbiased, as shown by the close correspondence of $E(\hat {\delta }_{(i)})$ and $\delta _{(i)}$ in the figure. A slight bias can be seen for the sparse scenario for hypothesis $i=2$. Note that under the sparse scenario, $\delta _{(1)}$ is nearly equal to $\mu _1= -6$, which is an outlying hypothesis and has rank 1 with high probability. This is an illustration of the desirable property of projack to *not* shrink outlying values, as these values largely maintain their original ranks in the re-ordered jackknife. Simulations for $m=10$ are qualitatively similar (not shown). We note that results do not depend much on the sample size, provided $n$ is large enough to permit numerous $K$-splits, because of the variance scaling implicit in each $z_i$. The MSE results are favorable for $i$ near 1 or $m$ (Figure 4 of Supplementary material). In summary, the projack is an effective bias reduction approach under a variety of scenarios. In Appendix D and File 2 of Supplementary material, we investigate small sample performance with $n=\{5, 10, 20\}$ for data that may be skewed. Our conclusions are that the projack remains useful for $n$ as small as 10, provided the data are not highly skewed.

### 2.5. Performance under the null for GWAS data

Here we illustrate the difficulty in providing unbiased rank effect estimates by analyzing null GWAS data. We used simulated association data using genotypes from the HapMap JPT$+ $CHB example dataset on the PLINK web site ($n=89$ individuals and $m=68\,757$ informative SNP markers). For each of 100 simulations, we generated random 45 cases and 44 controls as “phenotypes,” independently of genotypes. For this complete null scenario, we obtained effect size estimates (expressed as $\mu$ estimates) using BR-squared and the conditional likelihood approach for $c$ values of 1.96, 3.48, and 5.45, corresponding to $p$-value thresholds of 0.05, 0.0005, and $5\times 10^{-8}$, respectively. In addition, we implemented projack-5 and projack-10. An unbiased estimation procedure will produce average effect estimates of zero for all genomic features, and we computed $z$ as the Cochran-Armitage trend statistic for each SNP genotype vs. case–control status. Appendix E and Figure 5 of Supplementary material depict the average estimates produced by BR-Squared, projack-5, projack-10, and the conditional likelihood approaches. For extreme $i$, both BR-Squared and the conditional likelihood approaches exhibit considerable bias in the same direction of the original ranked statistics, except for the conditional likelihood when using the threshold $p\lt 5\times 10^{-8}$, as almost all values were shrunk to zero. However, it is difficult to recommend the conditional likelihood for such an extreme threshold, as later simulations show that it can shrink indiscriminately and “miss” features that are important but not genome-wide significant. The bias in BR-Squared was quite substantial, with average effect estimates of roughly $-2.0$ and 1.5 for $i=1$ and $i=m$, respectively. The projack approaches show almost no bias.

## 3. The independent projack

For a number of problems, a single vector of ${\textbf{z}}$ is observed. Without the original data, methods such as BR-Squared are not applicable, as the data cannot be subdivided for resampling. However, we can extend our projack to this setting by making the following observations. First, we show in Appendix F of Supplementary material that, under the idealized form of the data, for large samples the projack is well approximated by Algorithm 1.

This algorithm for the independent projack is not immediately obvious, and results from a consideration of a hypothetical matrix ${\textbf{C}}$ such that $r({{\textbf{C}}})={\textbf{z}}$, and approximate bivariate normality of the row means for the training and test portions. An important advantage to the independent projack is that $K$ no longer need to be an integer or a divisor of $n$, and the entire range $K\in (1,\infty )$ is available, although in practice values in the range 5–10 may be advisable.

**Algorithm 1.** The independent projack

1:	Simulate a vector ${\textbf{z}}^{\prime }={\textbf{z}}+ {\boldsymbol \gamma }$, where the $\{\gamma _i\}$ are drawn iid $N(0,1/(K-1))$.
2:	Compute a new vector ${\textbf{c}}=K {\textbf{z}}-(K-1){\textbf{z}}^{\prime }$, and reorder to create ${\textbf{d}}={\textbf{c}}_{(.)}$, where (importantly) the re-ordering is based on ${\textbf{z}}^\prime$.
3:	Repeat steps 1–2, averaging over many ordered simulated vectors ${\textbf{d}}$ to obtain $\hat {\delta }_{(.)}$.

We recommend performing the simulations at least 1000 times, in order to minimize stochastic variation. Each simulation step of the independent projack corresponds to a single random $K$-fold split of the projack, and so the independent projack is somewhat faster and uses much less memory (handling vectors rather than matrices).

### 3.1. The psoriasis example, continued

We return to the psoriasis example described in Section 1, using the independent projack and competing methods. For the independent projack, we augmented $z$ statistics for the observed “top” SNPs with additional artificial $m-10$ SNPs with each $z_i=\Phi (i/(m-9))$, and $m=480\,000$. For BR-Squared, we used the results as reported in Faye *and others* (2011). For the conditional likelihood approaches, we used $c$ values of 1.96, 3.48, and 5.45, corresponding to $p$-value thresholds of 0.05, 0.0005, and $5\times 10^{-8}$, respectively. Figure 3 shows the results of the analysis. The left panel (black dots) shows the naive estimates vs. replication odds ratios, which are all above the unit line. Both BR-Squared and projack-5 appear to overshrink somewhat for the SNPs of moderate effect, but the projack is less aggressive than BR-Squared for the SNPs of smallest effect. The curve in the right panel shows the projack squared error averaged across the 10 SNPs as a function of $K$. The error is minimized for $K=6$, which is also the minimum value in terms of mean absolute bias and median absolute bias (not shown). The plot also shows the results for the naive uncorrected estimates, BR-Squared, and the conditional likelihood (the result for $c=5.45$ is at 0.032, out of plotting range). In this example, projack dominates BR-Squared throughout the $K=5$–10 range, but the conditional likelihood for $p\lt 0.0005$ performs relatively well. Analysis of a recent genome-wide association scan for hypospadias-associated loci shows similar results (Appendix G of Supplementary material).

### 3.2. GWAS simulations

Here we decribe additional simulations, using 875 samples from the 1000 Genomes project, as described in Abdo *and others* (2015) and retaining SNPs with minor allele frequency $\ge 0.05$, for $\sim$1.41 million SNPs. The genotypes were adjusted for the top 20 principal components, to ensure that effects for putative causal SNPs would not carry across distant genomic regions due to population stratification. We simulated quantitative phenotypes $y$ according to a model in which some genotyped SNPs were selected as causal, and for the $j$th individual $y_j=\sum _{i \in {\rm causal}}\beta _i x_{ij}+ \epsilon _j$, where $\epsilon \sim N(0,1)$. The $\beta$ values were determined by first computing null standard errors for each causal SNP, selecting $\mu$ values as described below, and then computing each $\beta =\mu SE$. Two scenarios were considered: (i) a multi-SNP scenario with 20 causal SNPs, with six extreme SNPs having $\mu =\{-12, -8, -6, 6, 8, 12\}$, respectively, and the $\mu$ values for the remaining 14 SNPs uniformly spaced in the range $\{-2,2\}$; (ii) a few-SNP scenario in which only two SNPs were chosen to be causal, with true underlying $\mu =\{-7,7\}$.

For genotype data, due to the presence of linkage disequilibrium, SNPs nearby the causal SNPs will also exhibit association with the trait. For causal SNP $i$ and nearby SNP $i^\prime$, the effect size at $i^\prime$ is $\mu _{i^\prime }=r_{i,i^\prime } \mu _i$, where $r_{i,i^\prime }$ is the genotype correlation between the two SNPs. Thus for each causal SNP, we consider SNPs nearby (200 SNPs upstream and downstream) as also “alternative,” provided $r_{i,i^\prime }^2 >0.05$. Quantitative phenotypes were simulated 100 times for each of the two scenarios, with the genotypes held fixed, and thus representing realistic linkage disequilibrium structure. As a large proportion of the genotypes were imputed, BR-Squared could not be used. However, the conditional likelihood approach can be used, and here we used the same values of $c$ as used for the psoriasis data. The true $\boldsymbol {\delta }_{(.)}$ was obtained by averaging for each row of the $\boldsymbol {\mu }_{(.)}$ matrix, i.e., each column of $\boldsymbol \mu$ sorted according to the ${\textbf{z}}$ vector for each simulation. Even for the “few-SNP” scenario, $\delta _{(i)}$ can differ noticeably from zero for (say) $i=100$, largely due to the effect of linkage disequilibrium on SNPs nearby to the causal SNPs.

**Fig. 4.**
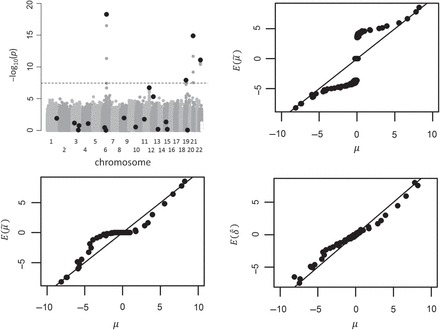
Simulations for the multi-SNP scenario with 20 causal SNPs among 1.4 milllion SNPs. Upper left: Manhattan plot of ${\rm -log}_{10}(p)$ for a single simulation, with causal loci indicated as large dots. Upper right: Over 100 simulations, comparison of $E(\tilde {\mu }_{(.)})$ vs. $\mu _{(.)}$ for the conditional likelihood using threshold $p\lt 0.05$, across all 1.4 million SNPs, for which an unbiased method would dots on the unit line. A vast majority of values are near the origin. Lower left: results from the conditional likelihood approach using $p\lt 0.0005$. Lower right: results for projack-$5$.

Figure 4 (upper left) illustrates the results of a single simulation from these data across the genome for the multi-SNP simulation, with the locations of the 20 causal SNPs indicated. The true $\delta _{(i)}$ was computed for each $i$, and many $>$20 of these values are far from zero, due to linkage disequilibrium capturing additional SNPs. These values are compared with expected estimates for two of the conditional likelihood approaches and projack-5 in the figure, showing that projack-5 is nearly unbiased. Table 2 shows the results in terms of mean-squared error averaged over collections of “top” SNPs (AMSE). For example, the top 20 entries consist of averaging estimates over simulations and over the SNPs with the 10 smallest (most highly negative) and 10 highest $z$ statistics. Let $\hat {\delta }_{i,s}$ denote the projack estimate for the $i$th ranked feature in simulation $s$ of a total $S$ simulations. Then ${\rm AMSE}=({1}/{S}) \sum _{s=1}^{S} ({1}/{T}) \{\sum _{i=1}^{T/2} (\hat {\delta }_{i,s}-\delta _i)^2 + \sum _{i=m-T/2}^{m} (\hat {\delta }_{i,s}-\delta _i)^2 \},$ and the same calculations were performed for the conditional likelihood. The results are shown in Table 2 for both scenarios. Note that the AMSE incorporates the effects of both bias, already shown to be low with projack, and variance. Projack-5 has the lowest AMSE in four of the six situations described, and is always among the top two (boldface). For the multi-SNP scenario and ranking the top 20 SNPs, the conditional likelihood with $p\lt 0.0005$ has the lowest MSE. However, for other situations, the same approach performs poorly, while the conditional likelihood with $p\lt 5\times 10^{-8}$ performs better. One difficulty lies in the fact that the conditional likelihood is not adaptive to the data at hand, while projack, based on perturbations from the observed data, is inherently and automatically adaptive.

**Table 2.** Mean squared errors for the various $\boldsymbol {\delta }_{()}$ estimation approaches

Method	Multi-SNP			Few-SNP		
	Top 20	Top 100	Top 200	Top 20	Top 100	Top 200
indep. projack-5	**3.23**	**1.03**	**0.53**	**2.87**	**0.97**	**0.53**
indep. projack-10	4.38	**1.37**	**0.72**	**3.90**	1.52	0.87
cond. like ($p\lt 0.05$)	2.81	10.66	12.12	10.73	14.50	14.45
cond. like ($p\lt 0.0005$)	**2.44**	6.60	5.35	9.07	9.42	6.99
cond. like ($p\lt 5\times 10^{-8}$)	6.66	2.42	1.25	4.09	**1.01**	**0.52**

Boldface indicates the two lowest values in each column.

## 4. Discussion

We have attempted to describe the important aspects of the ranked estimation problem. Our proposed solution is a frequentist general resampling method for prediction of ranked effects and estimation of normal means, without explicit smoothing or Bayesian inference. The projack is ready to be used as a low-bias estimator/predictor of ranked effects in large-scale multiple testing settings. Further work remains in fully understanding the tradeoffs between bias and variance, although choices of $K$ in the range of 5–10 appear to work well. When the original data are available, part of the appeal of projack lies in its use of the observed data correlation structure, and there is no inherent difficulty posed by datasets in which $m \gg n$. Extensions of projack may include analogues to penalized regression, as the shrinkage inherent in the approach could be applied to regression coefficients. However, considerable work would remain in choosing appropriate penalty conditions, especially in sparse coefficient settings.

## 5. Software

$R$ software is available upon request.

## Funding

This study is supported in part by NIH R01MH101819 and NSF DMS-1127914. Funding to pay the Open Access publication charges for this article was provided by institutional funds at North Carolina State University.

## Supplementary Material

Supplementary Data

Supplementary Data
