# Supplementary material for: The projack: a resampling approach to correct for ranking bias in high-throughput studies
Source: Biostatistics. 2015 Jun 3;17(1):54–64. doi: 10.1093/biostatistics/kxv022 (PMC4679068; doi:10.1093/biostatistics/kxv022)
Supplement: Supplementary Data [file kxv022supp_data1.pdf]

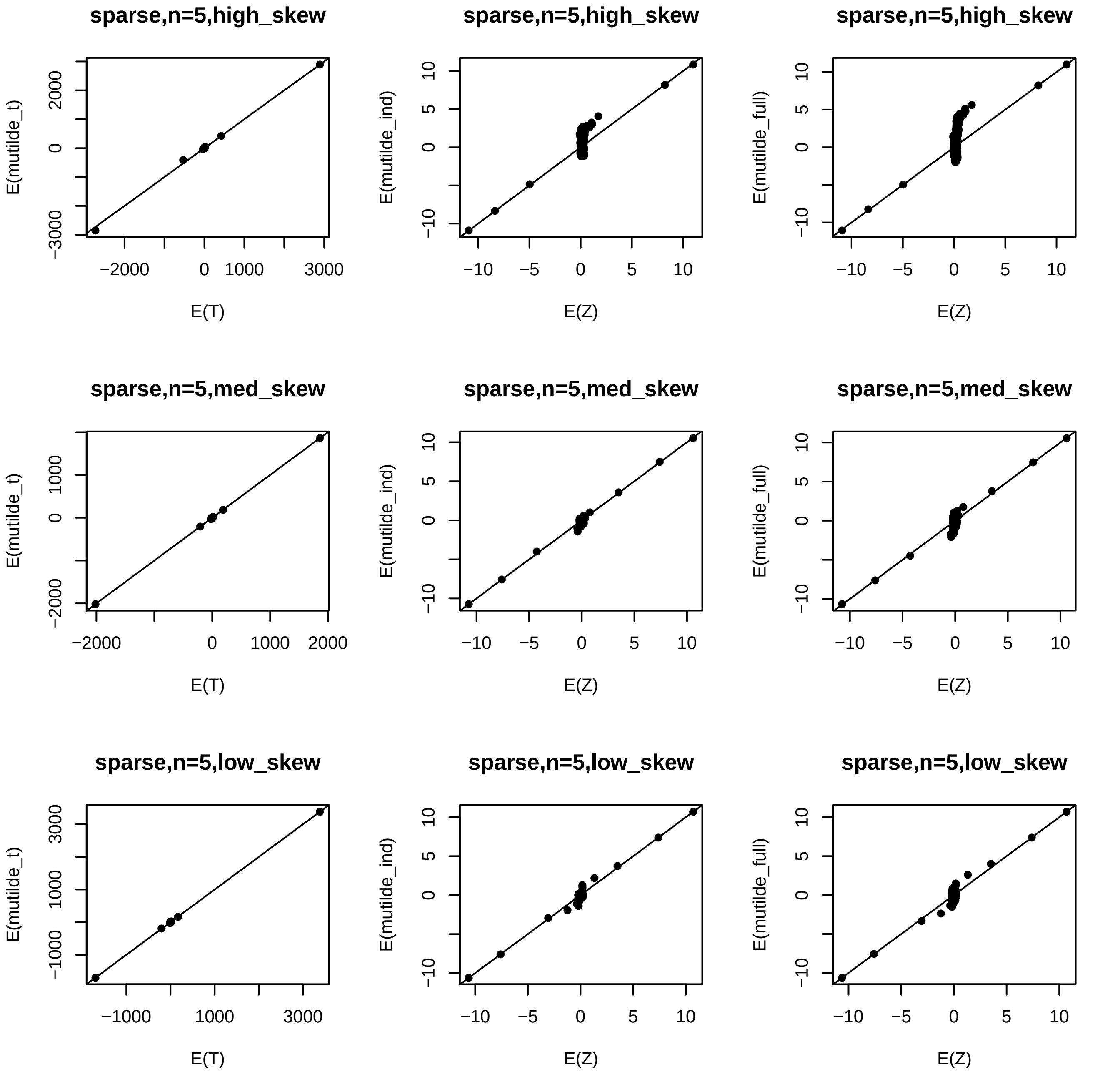
sparse,n=5,high_skew
sparse,n=5,high_skew
sparse,n=5,high_skew
E(mutilde_t)
E(mutilde_ind)
E(mutilde_full)
E(T)
E(Z)
E(Z)
sparse,n=5,med_skew
sparse,n=5,med_skew
sparse,n=5,med_skew
E(mutilde_t)
E(mutilde_ind)
E(mutilde_full)
E(T)
E(Z)
E(Z)
sparse,n=5,low_skew
sparse,n=5,low_skew
sparse,n=5,low_skew
E(mutilde_t)
E(mutilde_ind)
E(mutilde_full)
E(T)
E(Z)
E(Z)

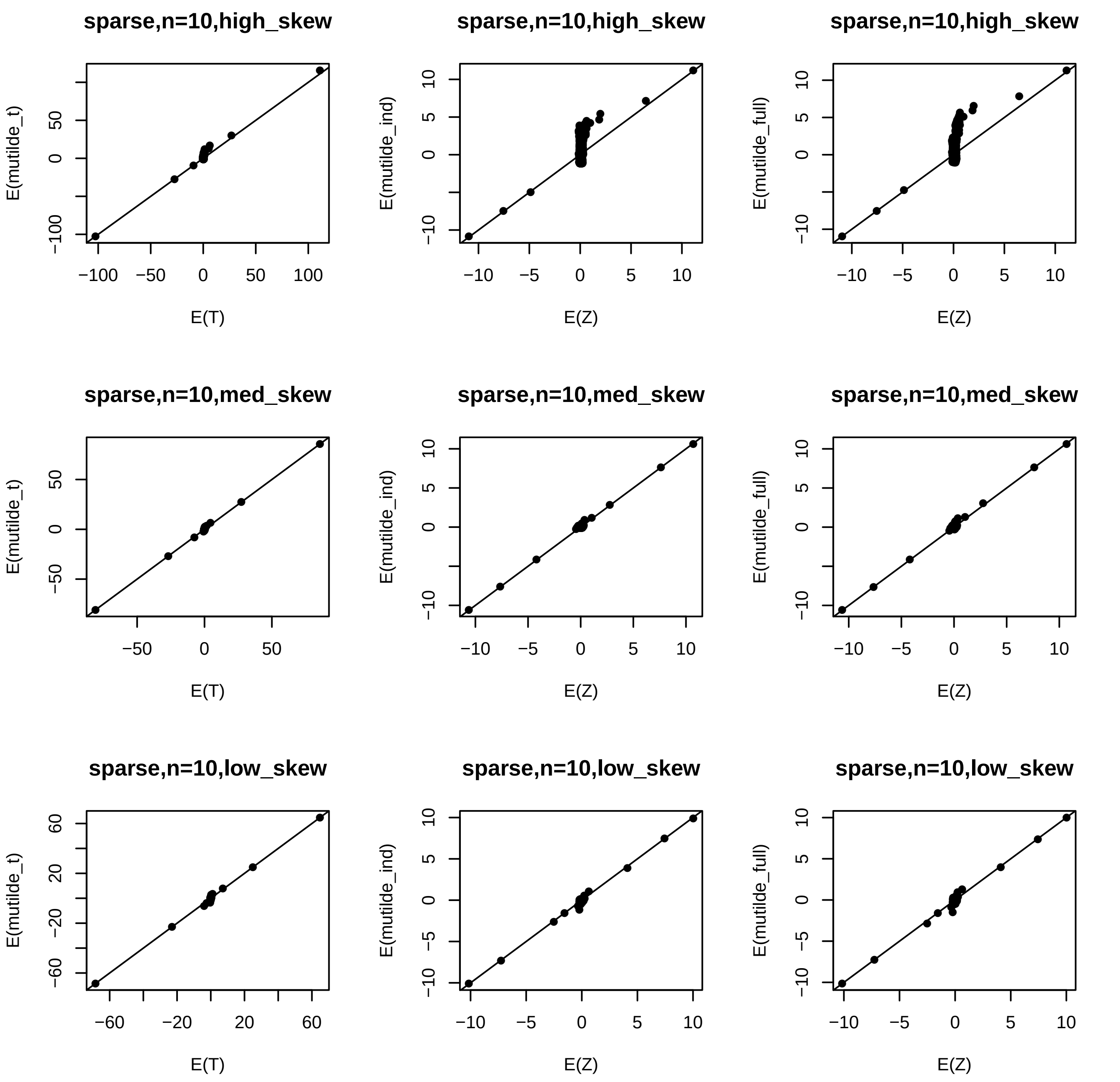
sparse,n=10,high_skew
E(mutilde_t)
E(T)
sparse,n=10,high_skew
E(mutilde_ind)
E(Z)
sparse,n=10,high_skew
E(mutilde_full)
E(Z)
sparse,n=10,med_skew
E(mutilde_t)
E(T)
sparse,n=10,med_skew
E(mutilde_ind)
E(Z)
sparse,n=10,med_skew
E(mutilde_full)
E(Z)
sparse,n=10,low_skew
E(mutilde_t)
E(T)
sparse,n=10,low_skew
E(mutilde_ind)
E(Z)
sparse,n=10,low_skew
E(mutilde_full)
E(Z)

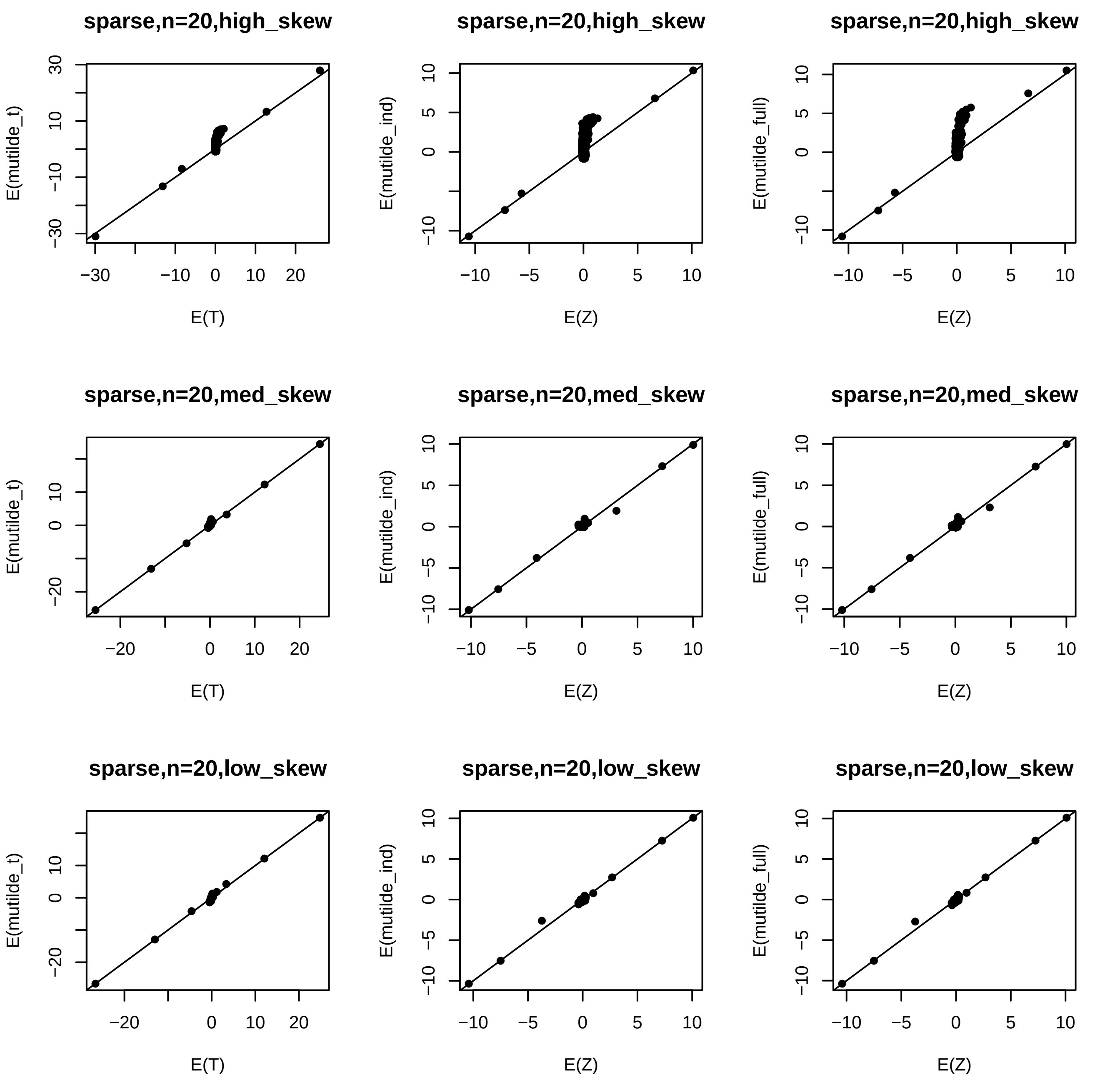

sparse,n=20,high_skew
E(mutilde_t)
30
10
−10
−30
−30
−10
0
10
20
E(T)
sparse,n=20,high_skew
E(mutilde_ind)
10
5
0
−10
−10
−5
0
5
10
E(Z)
sparse,n=20,high_skew
E(mutilde_full)
10
5
0
−10
−10
−5
0
5
10
E(Z)
sparse,n=20,med_skew
E(mutilde_t)
10
0
−20
−20
0
10
20
E(T)
sparse,n=20,med_skew
E(mutilde_ind)
10
5
0
−5
−10
−10
−5
0
5
10
E(Z)
sparse,n=20,med_skew
E(mutilde_full)
10
5
0
−5
−10
−10
−5
0
5
10
E(Z)
sparse,n=20,low_skew
E(mutilde_t)
10
0
−20
−20
0
10
20
E(T)
sparse,n=20,low_skew
E(mutilde_ind)
10
5
0
−5
−10
−10
−5
0
5
10
E(Z)
sparse,n=20,low_skew
E(mutilde_full)
10
5
0
−5
−10
−10
−5
0
5
10
E(Z)

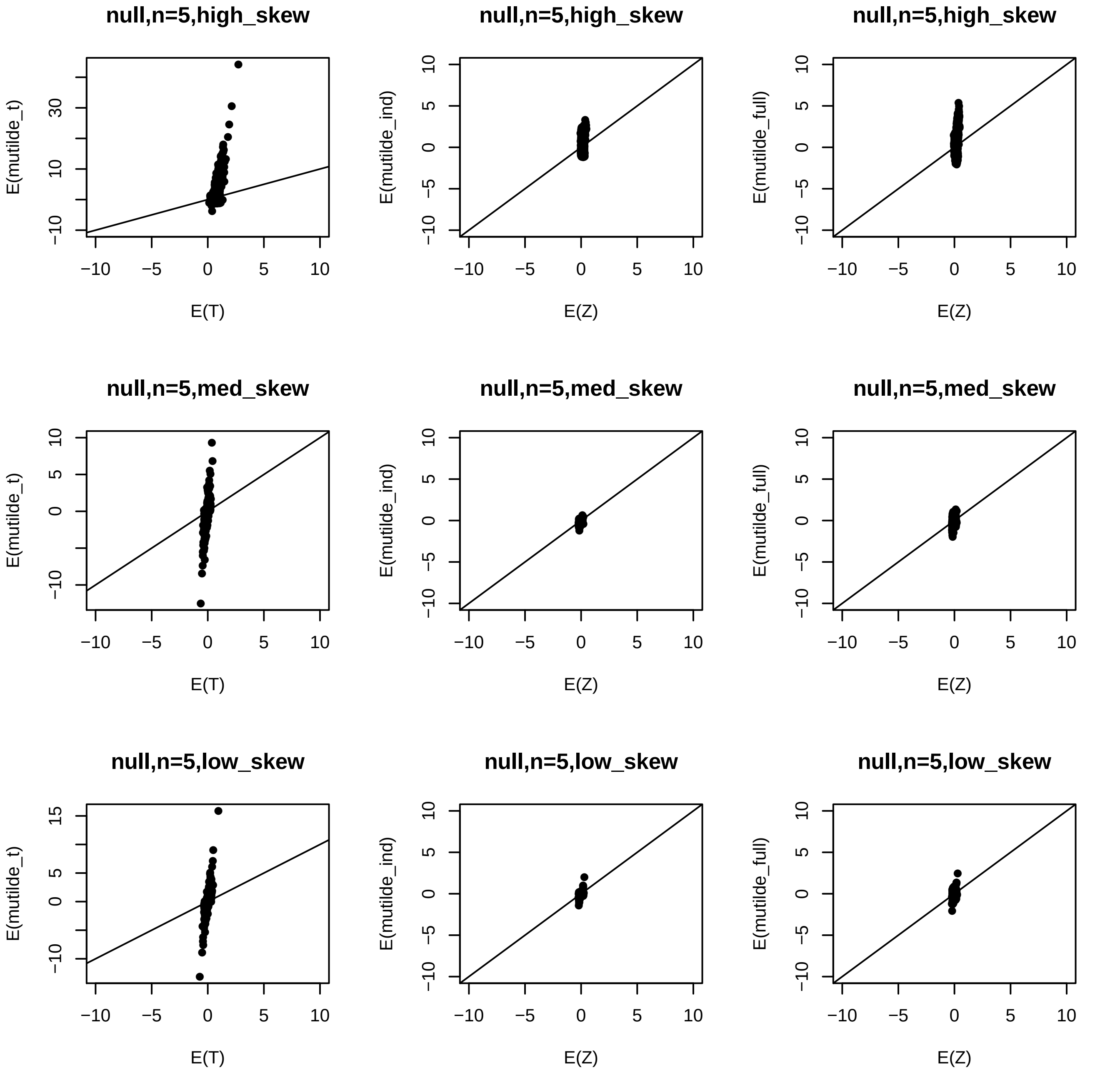
null,n=5,high_skew
E(mutilde_t)
E(T)
null,n=5,high_skew
E(mutilde_ind)
E(Z)
null,n=5,high_skew
E(mutilde_full)
E(Z)
null,n=5,med_skew
E(mutilde_t)
E(T)
null,n=5,med_skew
E(mutilde_ind)
E(Z)
null,n=5,med_skew
E(mutilde_full)
E(Z)
null,n=5,low_skew
E(mutilde_t)
E(T)
null,n=5,low_skew
E(mutilde_ind)
E(Z)
null,n=5,low_skew
E(mutilde_full)
E(Z)

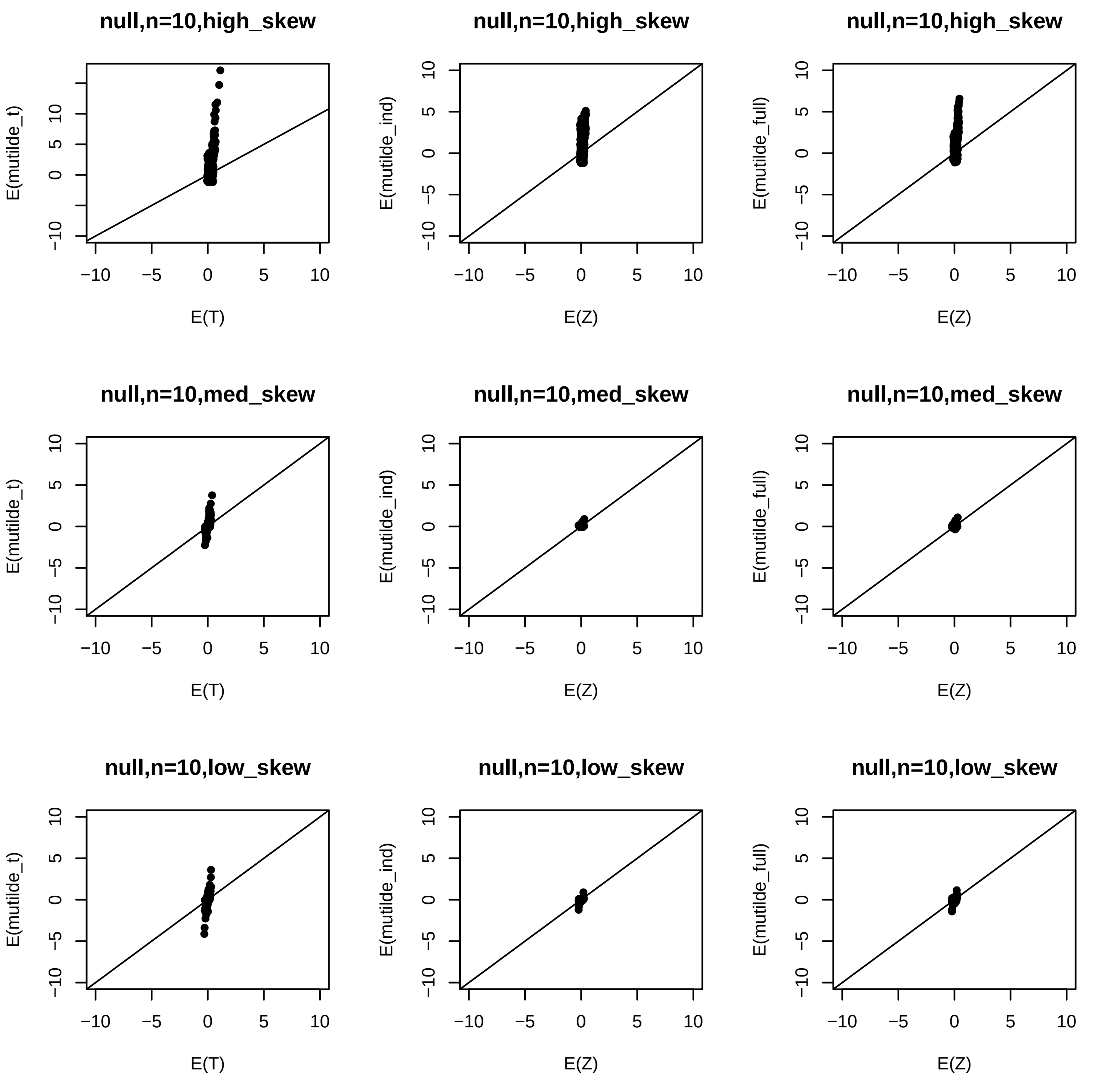
null,n=10,high_skew
E(mutilde_t)
E(T)
null,n=10,high_skew
E(mutilde_ind)
E(Z)
null,n=10,high_skew
E(mutilde_full)
E(Z)
null,n=10,med_skew
E(mutilde_t)
E(T)
null,n=10,med_skew
E(mutilde_ind)
E(Z)
null,n=10,med_skew
E(mutilde_full)
E(Z)
null,n=10,low_skew
E(mutilde_t)
E(T)
null,n=10,low_skew
E(mutilde_ind)
E(Z)
null,n=10,low_skew
E(mutilde_full)
E(Z)

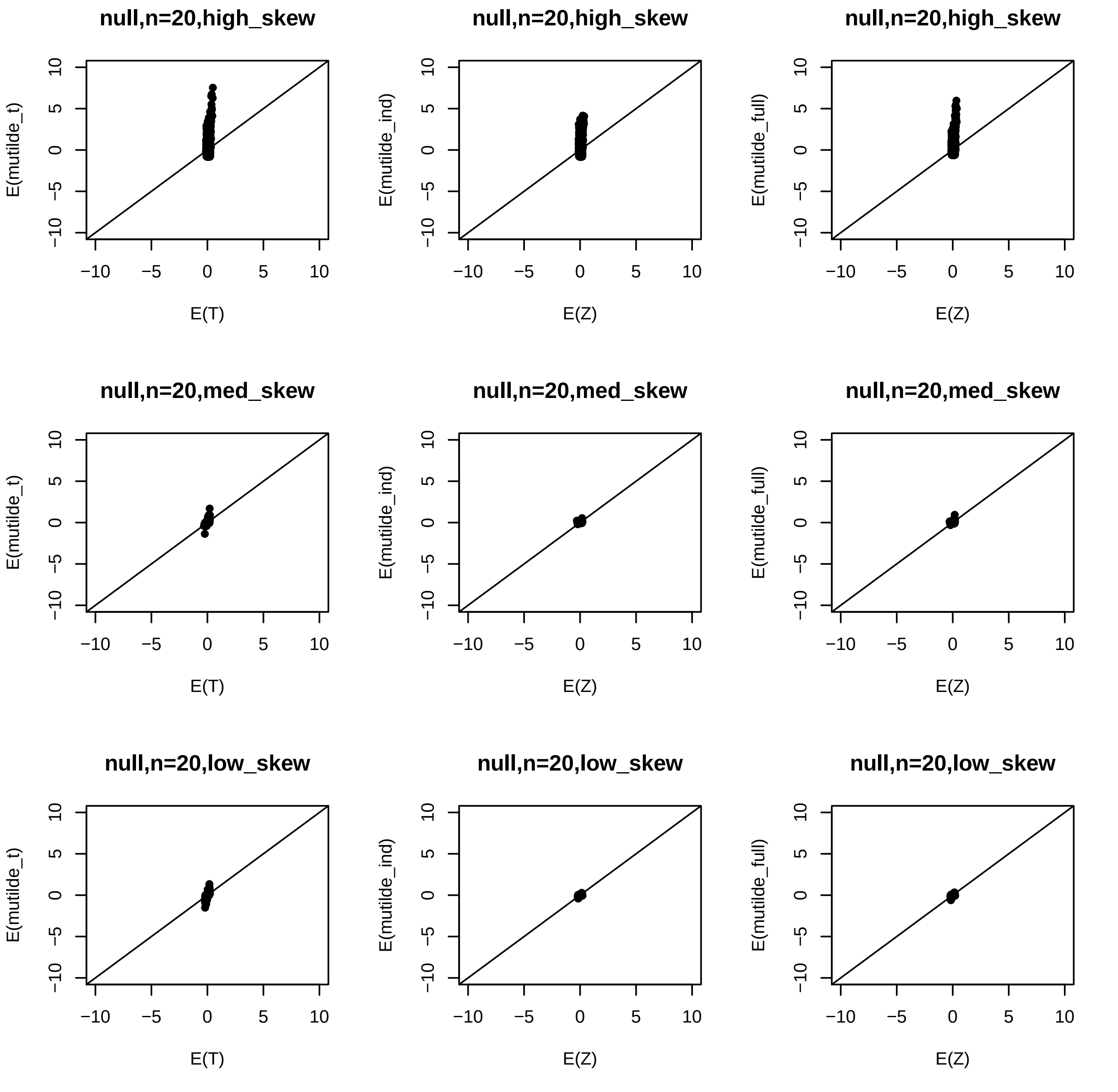
null,n=20,high_skew
E(mutilde_t)
E(T)
null,n=20,high_skew
E(mutilde_ind)
E(Z)
null,n=20,high_skew
E(mutilde_full)
E(Z)
null,n=20,med_skew
E(mutilde_t)
E(T)
null,n=20,med_skew
E(mutilde_ind)
E(Z)
null,n=20,med_skew
E(mutilde_full)
E(Z)
null,n=20,low_skew
E(mutilde_t)
E(T)
null,n=20,low_skew
E(mutilde_ind)
E(Z)
null,n=20,low_skew
E(mutilde_full)
E(Z)

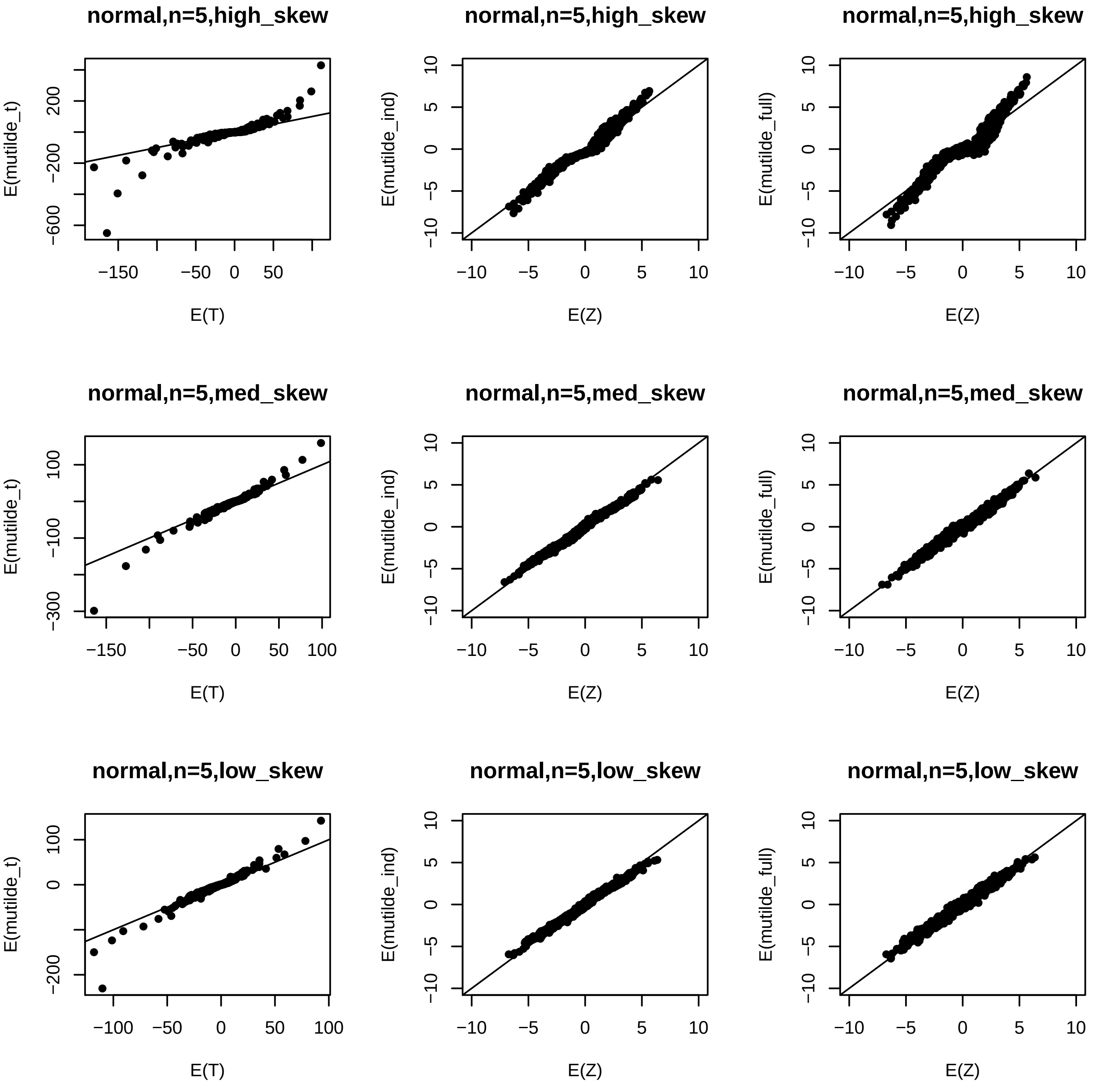
normal,n=5,high_skew
E(mutilde_t)
−600
−200
200
−150
−50
0
50
E(T)
normal,n=5,high_skew
E(mutilde_ind)
−10
−5
0
5
10
E(Z)
normal,n=5,high_skew
E(mutilde_full)
−10
−5
0
5
10
E(Z)
normal,n=5,med_skew
E(mutilde_t)
−300
−100
100
−150
−50
0
50
100
E(T)
normal,n=5,med_skew
E(mutilde_ind)
E(Z)
normal,n=5,med_skew
E(mutilde_full)
E(Z)
normal,n=5,low_skew
E(mutilde_t)
−200
0
100
−100
−50
0
50
100
E(T)
normal,n=5,low_skew
E(mutilde_ind)
E(Z)
normal,n=5,low_skew
E(mutilde_full)
E(Z)

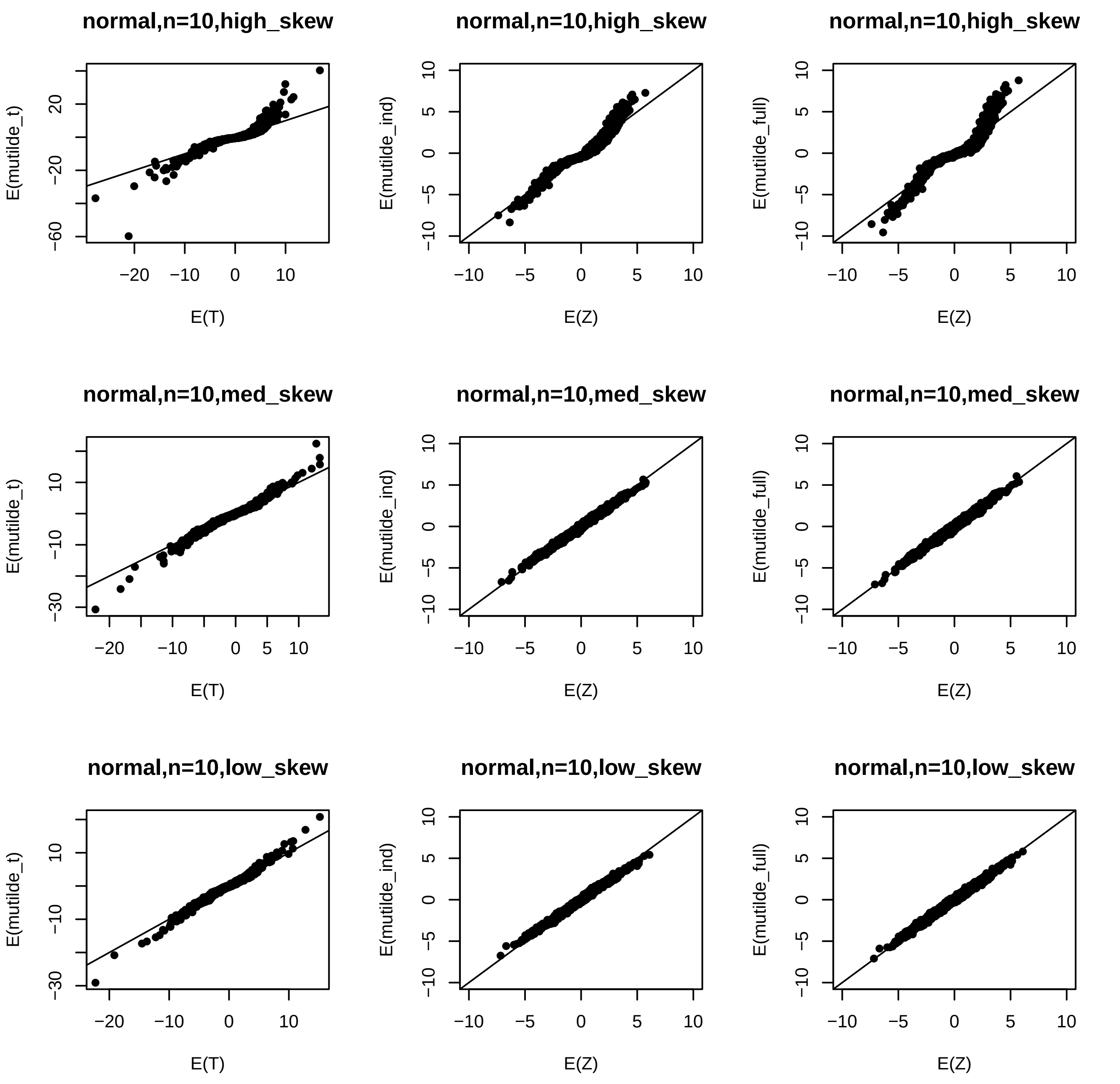

normal,n=10,high_skew
E(mutilde_t)
−60
−20
20
−20
−10
0
10
E(T)
normal,n=10,high_skew
E(mutilde_ind)
−10
−5
0
5
10
E(Z)
normal,n=10,high_skew
E(mutilde_full)
E(Z)
normal,n=10,med_skew
E(mutilde_t)
−30
−10
10
−20
−10
0
5
10
E(T)
normal,n=10,med_skew
E(mutilde_ind)
E(Z)
normal,n=10,med_skew
E(mutilde_full)
E(Z)
normal,n=10,low_skew
E(mutilde_t)
−30
−10
10
−20
−10
0
10
E(T)
normal,n=10,low_skew
E(mutilde_ind)
E(Z)
normal,n=10,low_skew
E(mutilde_full)
E(Z)

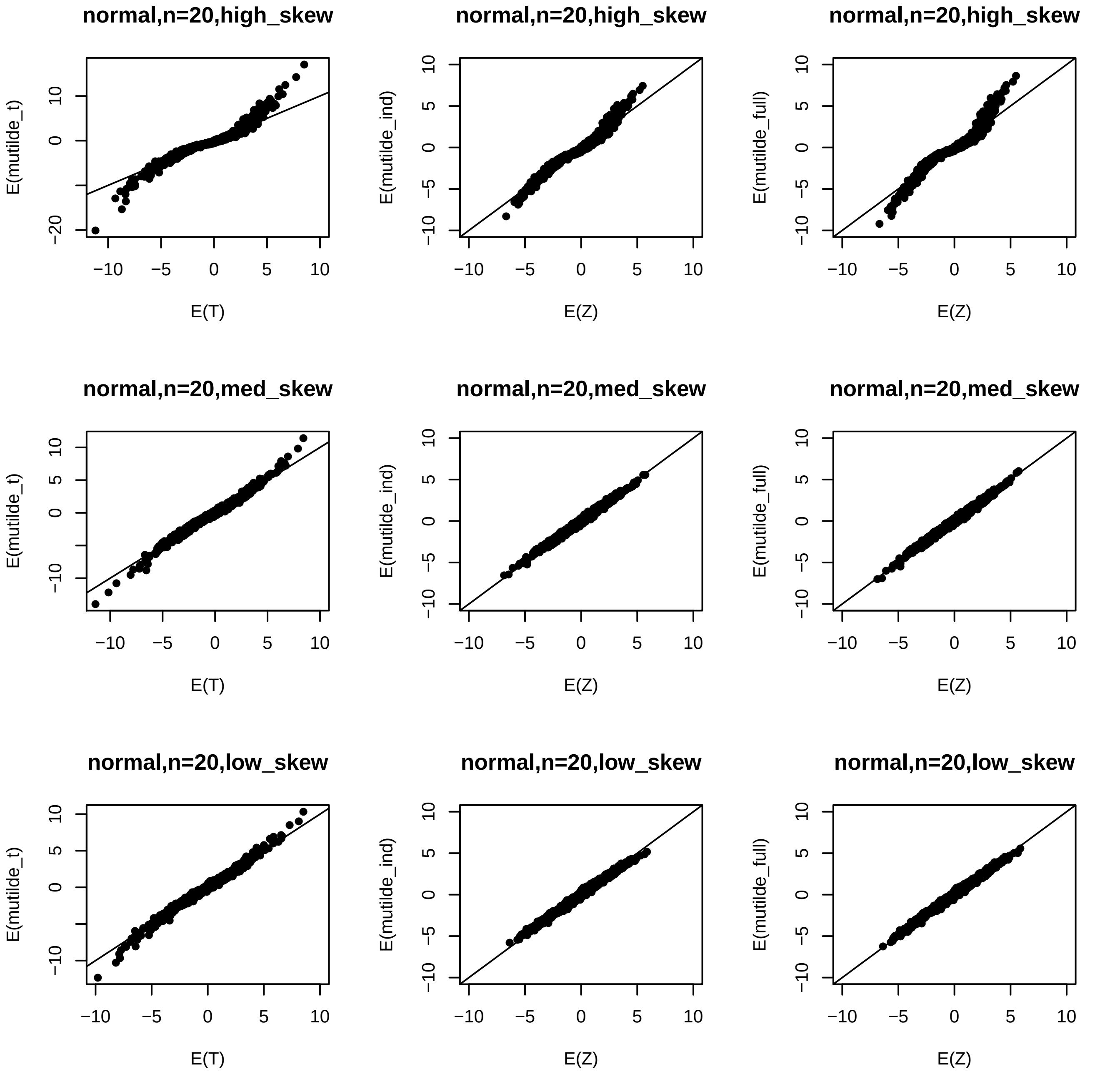

normal,n=20,high_skew
E(mutilde_t)
E(T)
normal,n=20,high_skew
E(mutilde_ind)
E(Z)
normal,n=20,high_skew
E(mutilde_full)
E(Z)
normal,n=20,med_skew
E(mutilde_t)
E(T)
normal,n=20,med_skew
E(mutilde_ind)
E(Z)
normal,n=20,med_skew
E(mutilde_full)
E(Z)
normal,n=20,low_skew
E(mutilde_t)
E(T)
normal,n=20,low_skew
E(mutilde_ind)
E(Z)
normal,n=20,low_skew
E(mutilde_full)
E(Z)
