# Supplementary material for: The projack: a resampling approach to correct for ranking bias in high-throughput studies
Source: Biostatistics. 2015 Jun 3;17(1):54–64. doi: 10.1093/biostatistics/kxv022 (PMC4679068; doi:10.1093/biostatistics/kxv022)
Supplement: Supplementary Data [file kxv022supp_data2.pdf]

SUPPLEMENTARY APPENDIX TO THE PROJACK: A RESAMPLING APPROACH TO CORRECT FOR RANKING BIAS IN HIGH-THROUGHPUT STUDIES

By Yi-Hui Zhou*, and Fred A. Wright*

North Carolina State University *

1. Appendix A. Jackknife pseudo-values.. We start with the basic concept of the jackknife pseudo-value (Salkind [5]), which in our context involves computing elements

$$c_{ij} = n\hat{\mu}_i - (n-1)\hat{\mu}_{i[-j]}.$$

Here $\hat{\mu}_i = z_i$, and we use $\hat{\mu}_{i[-j]} = z_{i[-j]}\sqrt{n/(n-1)}$. The $\sqrt{n/(n-1)}$ term is necessary to correct for the fact that μ_i is an artificial "parameter," and does not converge to a constant but tends to grow with n at rate $\sqrt{n}$. Thus the z-statistic, computed on $n-1$ observations of the submatrix, tends to be smaller by a factor $\sqrt{(n-1)/n}$, is corrected so the expectation matches with the overall μ reflective of the original matrix. The construction of a z statistic based on the full data may be viewed as approximately modeling $E(C_{ij}) = \mu_i$, $\text{var}(C_{ij}) = n$, so that $E(Z_i) = E(\sum_j C_{ij}/n) = \mu_i$, $\text{var}(Z_i) = 1$. However, $Z_{i[-j]} = \sum_{j' \neq j} C_{ij'}/\sqrt{(n-1)n)}$ where the denominator is necessary to maintain $\text{var}(Z_{i[-j]}) = 1$. Thus $E(Z_{i[-j]}) = \mu_i\sqrt{(n-1)/n}$, and $\mu_i = E(Z_{i[-j]})\sqrt{n/(n-1)}$.

We illustrate the concept using the HapMap Asian ancestry data used for teaching purposes in the PLINK software (Purcell *and others* [4]) (JPT+CHB, $n = 89$ individuals and $m = 68,727$ informative SNP markers). We simulated a single random vector y consisting of 35 0 values and 54 1 values, to emphasize that the pseudo values do not require balanced data. Using the ancestry (JPT vs. CHB) as a covariate, we performed logistic regression using the R glm function for the model $y \sim ancestry + SNP$ and produced z values using Wald statistics. The pseudo matrix was also created using the jackknife procedure described. Supplementary Figure 1 shows $r(\mathbf{C})$ vs. z for the first 1000 SNPs having minor allele frequency greater (MAF) than 0.1, showing that the procedure achieves the desired result. The results show greater departure for lower MAF, but inspection of the results indicates that lack of fit results from a large influence from individual heterozygote values, which are less of a problem for larger sample sizes.

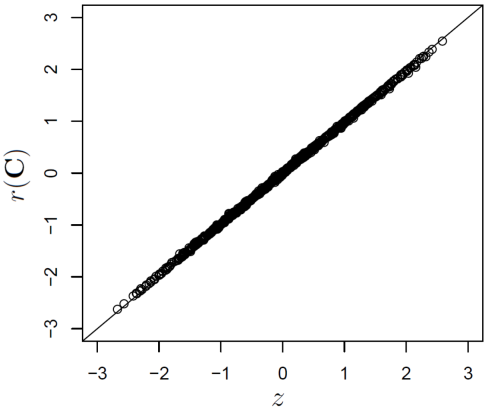

Supplementary Figure 1. *Pseudo-values created as described for logistic modeling of the HapMap JPT+CHB data have row means that are approximately the same as the original z-statistics.*

[3] and [6] describe conditions under which jackknife pseudo-values are asymptotically (large n) uncorrelated (for values within the same row of $\mathbf{C}$) and that the pseudo-value mean is asymptotically normal. In applications the pseudo-value mean is typically close to nz_i. Thus we treat the pseudo-values as if they retain basic moment properties from an idealized normal data structure, i.e. $E(C_{ij}) = \mu_i$, $\mathrm{var}(C_{ij}) = n$, and $\mathrm{corr}(C_{ij}, C_{i'j}) = \mathrm{corr}(Z_i, Z_{i'})$. These properties serve to justify approximate exchangeability for the reordered jackknife.

In general, computing the pseudo-matrix $\mathbf{C}$ can be computationally demanding, as it requires computing the overall z vector n times. However, we note that for most generalized linear models in common use and without nuisance covariates, the score statistics take the form $\sum_j x_{ij}y_j$, and thus after appropriate rescaling the values $x_{ij}y_j$ can be used for the elements of $\mathbf{C}$, thus requiring no leave-one-out computation in order to construct $\mathbf{C}$. For linear regression models, even in the presence of covariates, the standard (t) test statistic is proportional to $\sum_j x_{ij,\mathbf{z}}y_{j,\mathbf{z}}$ where the $\mathbf{z}$ signifies that each of genotype and phenotype are residuals, having been corrected for a covariate vector $\mathbf{z}$, and thus after covariate correction we have the same simple form (also see Kennedy and Cade, 1996). However, we note that the Potter (2005) logistic regression permutation approach performs covariate correction of the predictor of interest only, and then performs logistic regression using the residuals as a response, and reported accurate type I error for this approach.

Approximation to $\mathbf{C}$. The Kennedy and Cade (1996)-like approach of applying linear regression to residualized response and predictors, applied to covariate correction in generalized linear models, often appears to be a practically useful approach even in the absence of theoretical justification. We illustrate with a simple simulation here for the logistic regression context typically used in case-control studies. We consider case-control phenotype $\mathbf{y}$, SNP genotypes $\mathbf{X}$ and a covariate vector $\mathbf{z}$. For analysis of genotype-phenotype association after correction for $\mathbf{z}$, we compute $\sum_j (x_j - \hat{x}_j)(y_j - \hat{y}_j)$ where $\hat{y}_j$ is the fitted value of y_j given $\mathbf{z}_j$ after logistic regression of $\mathbf{y} \sim \mathbf{z}$, and $\hat{x}_{ij}$ the fitted genotype after linear regression of $\mathbf{x}_{i.}$ on $\mathbf{z}$. An approximate pseudo-value is immediately apparent from the summation form, but must be appropriately scaled in order to match the required variance characteristics of $\mathbf{C}$. For length-n vector $\mathbf{w}$ we define the scale operation $scale(\mathbf{w})$ as resulting in new values $(w_j - \bar{\mathbf{w}})/s_{\mathbf{w}}$, where $\bar{\mathbf{w}}$ is the sample mean and $s_{\mathbf{w}}$ the sample standard deviation of the vector $\mathbf{w}$. Then we compute $\mathbf{y}^{\ddagger} = scale(\mathbf{y} - \hat{\mathbf{y}})$ and for each i compute $\mathbf{x}^{\ddagger}_{i.} = \sqrt{n-1} scale(\mathbf{x}_{i.} - \hat{\mathbf{x}}_{i.})$, both operations which are extremely fast computationally. Finally, the result is the estimated $\mathbf{C}^{\ddagger} = \mathbf{X}^{\ddagger} * \mathbf{y}^{\ddagger}$, where '*' signifies the element-wise product $c^{\dagger}_{ij} = x^{\ddagger}_{ij} y^{\ddagger}_j$. Thus we can calculate $\mathbf{C}^{\ddagger}$ extremely quickly.

We illustrate here with a simulated example with $m = 10,000$, $n = 1000$, a single covariate $Z \sim N(0,1)$, and each X_{ij} is a binomial random variable with 2 trials and success probability $e^{\beta_{0i} + \beta_{1i} Z_j}/(1 + e^{\beta_{0i} + \beta_{1i} Z_j})$, with β_0 and β_1 each drawn from $N(0,1)$. The resulting genotypes have minor allele frequencies ranging from 0.025 to 0.5. Y_j is Bernoulli with success probability $e^{-1+Z_j}/(1 + e^{-1+Z_j})$. In this example, both Y and $\mathbf{X}$ are strongly correlated with Z for many SNPs, and thus with each other, although genotype and phenotype are conditionally independent given Z. Moreover, the case:control ratio is intentionally unbalanced ($P(case) \approx 0.29$) to avoid optimistic performance due to balanced $\mathbf{y}$. The left panel of Supplementary Figure 2 shows the result of applying the approximation, vs. the target z obtained as the maximum-likelihood Wald statistic from logistic regression. The approximation is very close, and far closer in simulations in which Z has a smaller effect on either $\mathbf{X}$ or $\mathbf{y}$ (not shown). This degree of close correspondence is of ultimate importance to projack, but we also illustrate here the approximate pseudo-values for the first column of $\mathbf{C}^{\ddagger}$ vs. the true pseudovalue computed using the jackknife method (right panel). Here the correspondence is slightly reduced, but close enough (correlation=0.996) that we expect the approxmation to have similar exchangeability properties as the more computationally intensive $\mathbf{C}$.

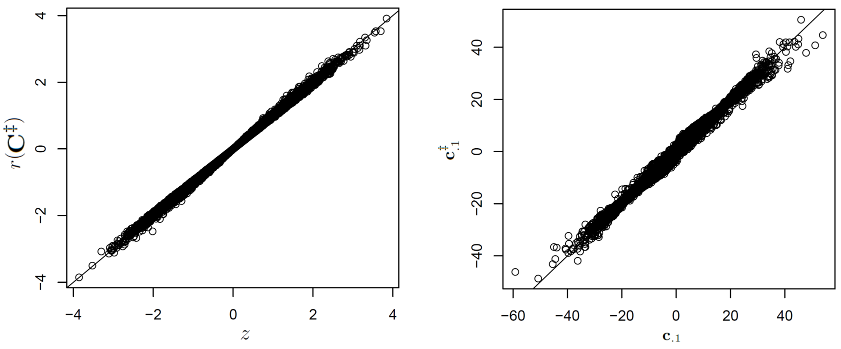

Supplementary Figure 2. *Results from the logistic regression example and approximate* $\mathbf{C}$. *Left panel: The row means of* $\mathbf{C}^{\ddagger}$ *vs. logistic regression Wald* z. *Right panel:* $\mathbf{c}^{\ddagger}_{.1}$ *vs. "true" pseudovalues* $\mathbf{c}_{.1}$.

Supplementary Table 1

Illustration of projack re-ordering.

Original $\mathbf{C}$				$\mathbf{C}_{(.)}$				$z_{(.)}$
0.04	0.78	2.20	0.18	-2.74	-0.73	-0.48	-0.39	-1.09
-0.37	-2.42	1.51	-1.91	-0.37	-2.42	1.51	-1.91	-0.80
-2.74	-0.73	-0.48	-0.39	-1.20	-3.25	1.97	1.85	-0.16
-1.20	-3.25	1.97	1.85	0.59	-0.51	1.48	0.97	0.63
0.59	-0.51	1.48	0.97	0.04	0.78	2.20	0.18	0.80

Ranks $(.)_{[-j]}$				Re-ordered data $\mathbf{D}$				$\hat{\delta}_{(.)}$
2	3	2	3	-0.37	-0.73	1.51	-0.39	0.01
3	2	3	4	-2.74	-2.42	-0.48	1.85	-0.95
4	1	4	2	-1.20	0.78	1.97	-1.91	-0.09
5	4	1	5	0.59	3.25	2.20	0.97	0.13
1	5	5	1	0.04	-0.51	1.48	0.18	0.30

2. Appendix B. Illustration of the re-ordered jackknife.. Here we illustrate the projack re-ordered jackknife. Supplementary Table 1 shows an initial matrix $\mathbf{C}$ and each of the subsequent steps in the re-ordered jackknife. The statistics z are computed as row mans of $\mathbf{C}$, and to obtain $\mathbf{D}$, each column is re-ordered according to the row means of the *remaining* columns, with the re-ranking indexes shown explicitly in the columns $Ranks_{(.)[-j]}$.

3. Appendix C. Projack bias and MSE performance for a variety of scenarios. For $n = 50$ and $m = 100$, we consider a variety of $\boldsymbol{\mu}$ vectors: (a) the null scenario where all $\mu_i = \mu_0$, and without loss of generality we use $\mu_0 = 0$; (b) a "sparse" scenario in which all but three hypotheses are null,

$\boldsymbol{\mu} = \{-6, -3, 0, ..., 0, 2\}$; (c) $\boldsymbol{\mu}$ exactly following a standard normal empirical cdf, i.e. $\mu_i = \Phi^{-1}(i/(m+1))$ for standard normal cdf Φ; (d) the "box"-shaped uniform $\boldsymbol{\mu}$ where the μ_i are equally spaced on the interval $[-2, 2]$. For each of the scenarios, we performed 1000 simulations, using $\Pi = 50$ splits. For each simulated matrix $\mathbf{C}$, we used two choices of K (5 and 10). In addition, we performed separate simulations in which the rows of $\mathbf{C}$ were uncorrelated with $\rho = 0$, or were moderately correlated with common $\rho = 0.4$. The results are shown in Supplementary Figure 3, and show that both projack-5 and projack-10 are nearly unibased for all i. A modest discrepancy between $\delta_{(i)}$ and $E(\hat{\delta}_{(i)})$ occurs in the sparse scenarios for $i = 2$.

For each fixed i, we also investigated the mean-squared error (MSE) of the naive $z_{(i)}$ vs. projack-5 and projack-10 over the simulations. For extreme i near 1 or m, the MSE is almost always superior to that of $z_{(.)}$. Near $i = m/2$, the MSE tends to be smaller for $z_{(.)}$. To understand this phenomenon, it is helpful to consider that the median (z_{50} when $m = 100$) in all the simulations is very near zero, due to symmetry, and thus has small variance. (As an extreme approach, one might estimate all $\delta_{(i)} = 0$, which would have small variance, but be a very poor estimator outside of the middle region.) In contrast, projack attempts to "find" a low-bias estimator empirically, even for ordered z values near the median, and the re-ranking increases variability. However, we are most interested in extreme i, so the bias-variance tradeoff for projack seems quite favorable.

4. Appendix D. Small-sample performance. The independent projack relies heavily on the normal assumption $z_i \sim N(\mu_i, 1)$, which for large sample sizes is often a reasonable approximation using central limit theorem arguments. The full-data projack does not technically rely on normality, but for small sample sizes the pseudomatrix construction may be suspect. Moreover, skewness in the data can have a greater effect for small sample sizes. To further investigate the behavior of projack under small sample sizes, we simulated small-n datasets under a variety of scenarios.

Here we fixed $m = 1000$ and simulated Y values as $Y \sim \chi^2_f$, and the degrees of freedom η was chosen from among $f \in \{1, 5, 20\}$, corresponding to high skew, medium skew, and lower skew scenarios. The effect sizes were determined by simulating an m-length vector $\boldsymbol{\beta}$, chosen to correspond to approximate $\boldsymbol{\mu}$ vectors as described below. Matrices $\mathbf{X}$ were determined using $X_{ij} = \beta_i y_{ij} + \epsilon_{ij}$, where each ϵ_{ij} was simulated as $(A_{ij} - \eta)/\sqrt{2\eta}$ and the A values simulated from χ^2_η, matching the skewness of the y values. Sample sizes of $n = \{5, 10, 20\}$ were used. Generation of the effect sizes and determining $\boldsymbol{\delta}_{(.)}$ values required some forethought. In order to judge the "pure"

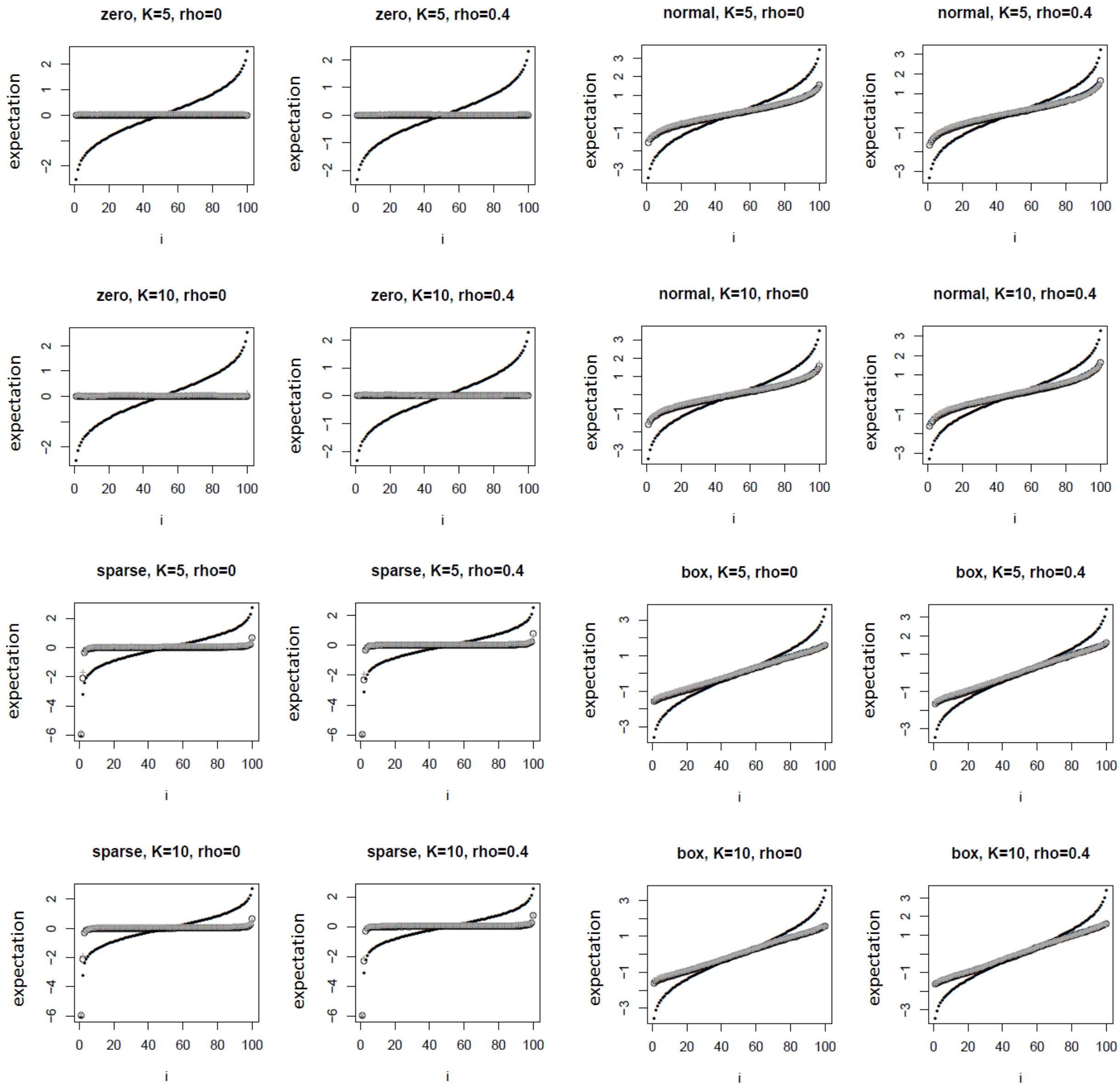

Supplementary Figure 3. *For each of the four scenarios described in the text (zero, sparse, normal, box), the values* $E(z_{(i)})$ *for each* i *are shown as black dots, and the true* $\delta_{(i)}$ *shown as open circles. The expectations of projack-5 and projack-10 are shown as grey crosses, and in almost all instances coincide nearly perfectly with* $\boldsymbol{\delta}$.

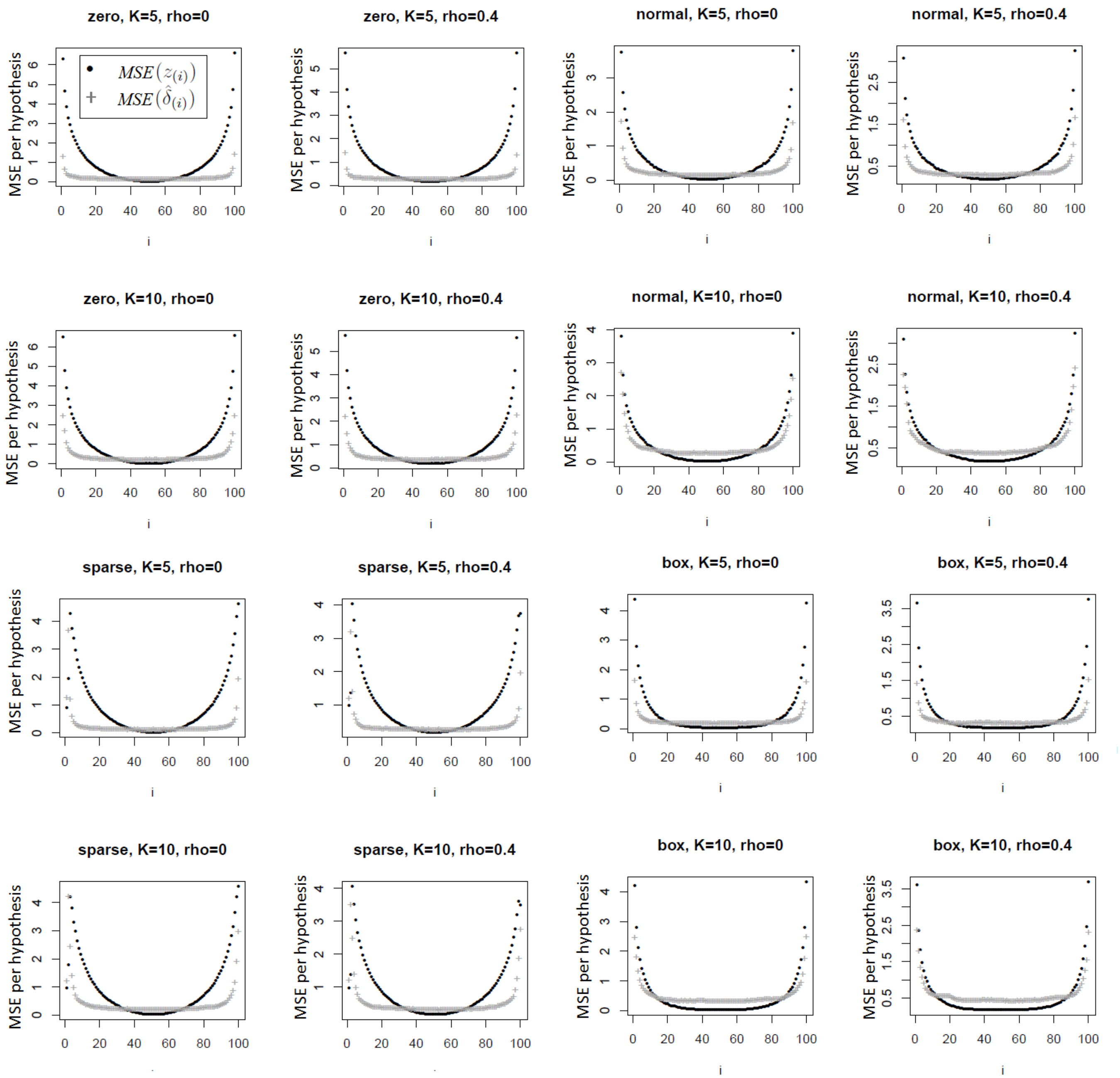

Supplementary Figure 4. *For each of the four scenarios described in the text (zero, sparse, normal, box) and the choices of* K *and correlation structures, the values* $MSE(z_{(i)})$ *for each* i *are shown as black dots, and the projack* $MSE(\hat{\delta}_{(i)})$ *shown as as grey crosses.*

effect of varying sample size, it is most instructive to choose $\boldsymbol{\mu}$ vectors that are invariant to the sample size (which is not true if one fixes the effect sizes and varies n). However, for small sample sizes the z statistics are not truly normal, and thus each $\mu_i = E(Z_i)$ must be determined by simulation, where here Z_i should be interpreted as whichever statistic is being used, e.g. a t-statistic or Wald statistic. We reasoned that the most stable approach to "achieving" a target $\boldsymbol{\mu}$ is to first compute the true correlation $\rho_i = \text{corr}(X_{i.}, Y)$, and then to compute the Fisher transformation f, where $\mu_i = f(\rho_i) = \sqrt{n-3}\log((1+\rho_i)/(1-\rho_i))$. For normal data (either X or Y), the Fisher transformation is often used to provide accurate testing for correlation coefficients $r = \text{corr}(X, Y)$, and also provides variance stabilization, such that $\text{var}(f(r_i)) \approx 1$. Its performance is not guaranteed for skewed data, but in our simulations appear to work well. Finally, the remaining task is to choose each β_i to satisfy the required ρ_i, which we perform by solving for the true correlation and the inverse Fisher transformation. We simulated $\boldsymbol{\mu}$ values according to three scenarios: the sparse scenario, where $\boldsymbol{\mu} = \{-10, -7, -4, 0..., 0, 4, 7, 10\}$, the zero scenario where each $\mu_i = 0$, and the normal scenario where $\mu \sim N(0, 2)$. Once $\boldsymbol{\mu}$ and therefore $\boldsymbol{\beta}$ was determined, for each simulation setting we generated 100 datasets, computing (i) the t statistics based on normal-assumption linear regression of y on each row of X, (ii) the Fisher-transformed statistic $z_i = f(r_i)$ where r_i is the sample Pearson correlation $\text{corr}(\mathbf{x}_{i.}, \mathbf{y})$. Then we computed each $\mu_{i,t}$ as the mean of the t statistic and $\mu_{i,z}$ as the mean of the Fisher z statistic. Finally, $\delta_{(i)t} = E(\mu_{(i)t})$ and $\delta_{(i)z} = E(\mu_{(i)z})$ values were computed by reordering the corresponding $\boldsymbol{\mu}$ values according to the statistics within each dataset and taking expectations for each (i).

For each dataset, we computed projack-5 estimates in three ways: (i) the independent projack, using the t statistics, (ii) independent projack using the z statistics, and (iii) the full-data projack using each $x_{ij}y_j$ as data elements of $\mathbf{C}$, appropriately scaled so that $\sum_j(x_{ij}y_j)$ would have mean 0 and variance exactly 1 under the null hypothesis of no association. We observed that even for $n = 20$, the pseudo-values could have row means differing noticeably from the z values, so each row of pseudo-values was adjusted by a constant to ensure $\sum c_{ij}/n = z_i$.

The results for the 27 scenarios (3 skewness levels)$\times$(3 sample sizes)$\times$(3 $\boldsymbol{\mu}$ settings) are shown in Supplementary File 2 as plots for each of the expected projack estimates vs. true $\boldsymbol{\delta}$. Examination of the sparse scenario indicates that the strategy for selecting $\boldsymbol{\beta}$ values approximately produced the target $\boldsymbol{\mu}$. For example, under that scenario we expect that $E(Z_{(1)})$ should be near $\mu_1 = -10$, because the μ value is extreme and isolated, and the plots

show that this is the case, even for $n = 5$ under high skew. Our procedure for selecting effect sizes was intended only to ensure that the results would be easily interpreted across varying sample sizes, and regardless, the bias-reducing success of projack is measured in terms of how well the values adhere to the unit line in each plot. A few overall observations appear relevant. Under the sparse scenario and small sample size, the extreme μ values produce such extreme t statistics that the projack appears to work well, but for larger sample sizes the reduced range of observations highlights that the group of hypothesis near the center are not estimated as well, especially in the high-skew setting. A similar observation holds for the z statistics under high skew. In general, the results for z statistics appear to be better than for t, but for very small sample sizes this effect may stem from the inappropriateness of using ordinary projack, which assumes that the variance of the statistics is always 1, to the small-sample t situation. For example, the variance of a central t-statistic for $n = 5$ is 3.0, and perhaps a modified independent projack could account for this increased variance by using larger variance in its perturbations. The Fisher-transformed z values, however, approximately stabilize the variance to 1, and thus the results shown can be considered a fair test of the performance of projack. Under medium or lower skew, either full-data or independent projack appears to work well, even for $n = 5$, but the high skew setting remains challenging even for $n = 20$. As would be expected, the performance for all scenarios improves as n increases.

It is worth noting that the χ^2_1 distribution is extremely highly skewed, and even the "low" skew distribution χ^2_{20} can be still be seen as noticeably skewed by examining its density. From our results, we suggest that the projack remains useful for n as low as 10 as long as $\mathbf{X}$ and $\mathbf{y}$ are not highly skewed, perhaps with a choice of z statistic to better stabilize the variance than a t-statistic. Such stabilization is not always available, however, e.g. with generalized linear models or for censored data. For $n \geq 20$, the results suggest that the projack is useful even for moderately skewed data, and in any event will certainly be much better than the naive approach of using $z_{(.)}$ directly.

5. Appendix E. Additional results on bias and mean-squared error under the complete null. The constant μ scenario, where $\delta_{(i)} = \mu_0$ for all i because each $\mu_i = \mu_0$, is an important special case. We note that for projack the bias performance will be identical for any μ_0. However, the conditional likelihood approach is inherently based on testing, so we illustrate here using the complete null scenario $\delta_{(i)} = 0$ for all i. Using the HapMap/PLINK Asian ancestry data described in the main text, we performed null simula-

tions using simulated "case/control" phenotype y consisting of 45 cases and 44 controls. Due to the time involved in computing BR-squared, we performed 10 null simulations, and for projack-5 used 10 simulations to form a fair comparison. All other methods used 100 simulations.

Supplementary Figure 5 (top panels) shows the results for the top 50 SNPs in each direction. Projack-5 is nearly unbiased, while ordered z is of course highly biased. BR-squared shows considerable bias in the direction of $z_{(.)}$, although we are uncertain as to the reason the bias seems more extreme for negative than for positive values. The condition likelihood using p-value threshold 0.0005 is highly biased, but the bias rapidly decreases as i increases from 1 to 50 or for i decreasing from m to $m - 50$. This drop in bias for the conditional likelihood corresponds to hypotheses that are rarely rejected, and so values of zero (which happen to be correct, because all $\mu_i = 0$) are substituted for the conditional likelihood. For this reason, the conditional likeihood using $p < 5 \times 10^{-8}$ has essentially perfect performance, as it is rare for any hypothesis to be rejected (not shown to avoid overplotting with projack). However, using such an extreme threshold makes the conditional likelihood of little use when dealing with data with small effect sizes. The conditional likelihood for $p < 0.05$ is essentially identical to using $z_{(.)}$, as almost all of the top 50 SNPs in each direction have $p < 0.05$ under every simulation.

The bottom panels of the figure show the mean-squared error (MSE) results. The top 150 results in each direction are shown, to better understand the "decay" in MSE. Again, projack-5 dominates the other procedures. We reiterate that the coniditonal likelihood with a very significant threshold (e.e. $P < 5 \times 10^{-8}$) will always perfom well in a null scenario, as it will, with high probability, declare no SNPs to be signifcant. However, such an extreme choice, while useful for the top SNPs when many SNPs are significant, is practically useless when only a few SNPs are significant, or when a large number of SNPs are nearly but not quite significant, but for which low-bias effect size estimates are still needed.

6. Appendix F. The independent projack. The projack can be extended to the problem where a single vector of independent $\mathbf{z}$ is observed. Conceptually, we envision the statistics as having arisen from a hypothetical matrix $\mathbf{C}$, with independent rows, elements with variance n, and $r(\mathbf{C}) = \mathbf{z}$. It is simple to simulate such a matrix with the requisite properties by starting with elements $B_{ij} \sim N(0, n)$ to populate a matrix $\mathbf{B}$, and then computing $\mathbf{C} = \mathbf{B} - r(\mathbf{B}) + \mathbf{z}$. Here we find $r(\mathbf{C}) = \mathbf{z}$. After this construction, we could perform projack-K on $\mathbf{C}$ to obtain $\hat{\boldsymbol{\delta}}$. A single $\mathbf{C}$ will, for sufficiently large

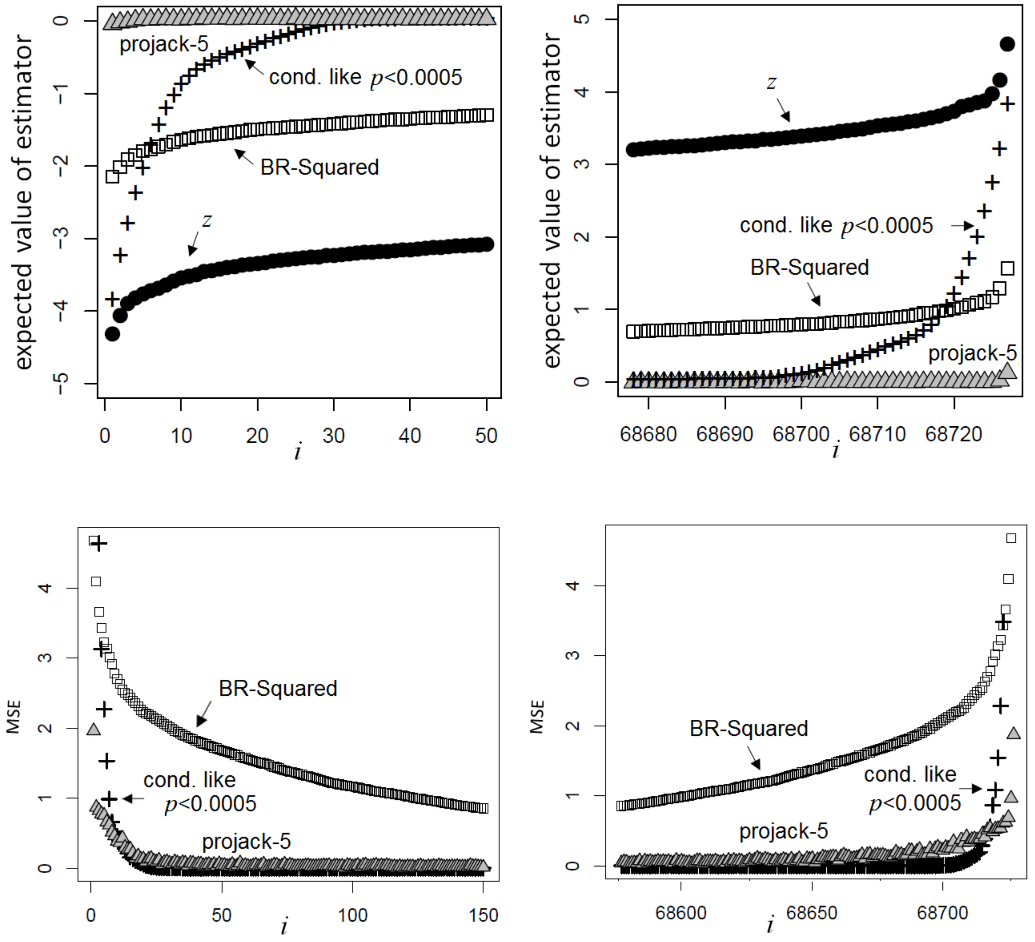

Supplementary Figure 5. *Top panels: The complete null scenario, HapMap/PLINK example data, top 50 SNPs in each direction shown (top left and top right) . The bias under the null is illustrated by examining, for each simulation, the top 50 SNPs in each direction and averaging the resulting estimates. A perfect method would give expected values of zero throughout. Bottom panels: Mean squared error for top 150 SNPs in each direction. All methods show increased MSE near the extremes $i = 1$ or $i = m$, but projack dominates throughout.*

Π (number of K-splits), produce an essentially fixed $\hat{\boldsymbol{\delta}}$.

The above concept satisfies the data structure for projack, but we argue here that simulating a full matrix $\mathbf{C}$ is unnecessary. First, we note that the expected behavior of projack for "large" n, or for that matter any n that is divisible by K, is identical to using $n = K$ for appropriately averaged data and for a fixed k-split. To see this fact, we follow the steps of Section 2.3, partitioning the columns of $\mathbf{C}$ into subsets J_k, and obtaining $\mathbf{C}^\dagger$. The row means using only the training columns of $\mathbf{C}$ are identical to $r(\mathbf{C}^\dagger_{[-K]})$, and therefore these row means have variance $K/(K-1)$, i.e., not dependent on n. Thus we will drop the "†" notation and assume $\mathbf{C}$ is $m \times K$, and still use k as the column index. Using the idealized assumed normal data structure, not conditional on the observed $\mathbf{z}$, each $C_{ik} \sim N(\mu_i, K)$. Defining $Z_i' = \sum_{k=1}^{K-1} C_{ik}/(K-1)$, we have $\mathrm{var}(Z_i') = K/(K-1)$, and $\{Z_i', Z_i\}$ is bivariate normal with $E(Z_i') = E(Z_i) = \mu_i$, and $\mathrm{corr}(Z_i', Z_i) = \sqrt{(K-1)/K}$. A comparison of the means and marginal variances shows that the relationship can be equivalently expressed $Z_i' = Z_i + \Gamma_i$, where $\Gamma_i \sim N(0, 1/(K-1))$. The remaining task is to condition on $Z_i = z_i$ for all i, to match the observed data. It is clear from the regression expression that $Z_i'|z_i \sim N(z_i, 1/(K-1))$. Notably, $Z_i'|z_i$ is (a) the basis for re-ordering rows in projack, and (b) not dependent on μ_i.

The implication of the above argument is as follows. Instead of simulating a matrix $\mathbf{C}$, with the attendant computational overhead, we can instead simulate a vector $\mathbf{z}' = \mathbf{z} + \boldsymbol{\gamma}$, where the $\{\gamma_i\}$ are drawn iid $N(0, 1/(K-1))$. $\mathbf{z}'$ is serving the role of the $n - n/K$ columns used for re-ordering in $\mathbf{C}$. To serve the role of the held-out n/K portion, we simulate a new vector $\mathbf{c} = K\mathbf{z} - (K-1)\mathbf{z}'$, and reorder to create $\mathbf{d} = \mathbf{c}_{(.)}$, where the re-ordering is based on $\mathbf{z}'$, because in the ordinary projack, re-ordering is based on the training set. A single simulation is analogous to performing projack-K on $\mathbf{C}$ for one re-ordering step, and it is easy to perform repeatedly, averaging over many simulated vectors $\mathbf{d}$ to obtain $\hat{\boldsymbol{\delta}}$. We call this approach, described as algorithm 1 in the main manuscript, the *independent projack*. An important advantage is that K need not be an integer, so that the entire stabilization range $K = (1, \infty)$ can be explored. An additional advantage is that the computation of standard errors is simplified. For an estimated $\hat{\boldsymbol{\delta}}$, many instances of $\mathbf{z}$ are simulated, and corresponding variability estimates obtained. Although the independent projack is based on simulation, it is computationally efficient and can be used for large m.

An illustration of the independent projack is shown in Supplementary Figure 5. A matrix $\mathbf{C}$ with $m = 100$, $n = 500$ was simulated with elements $C_{ij} \sim N(\mu_i, n)$. Here $\boldsymbol{\mu} = \{-6, -5, \mu_3, ..., \mu_{m-2}, 3, 4\}$, where the unspecified

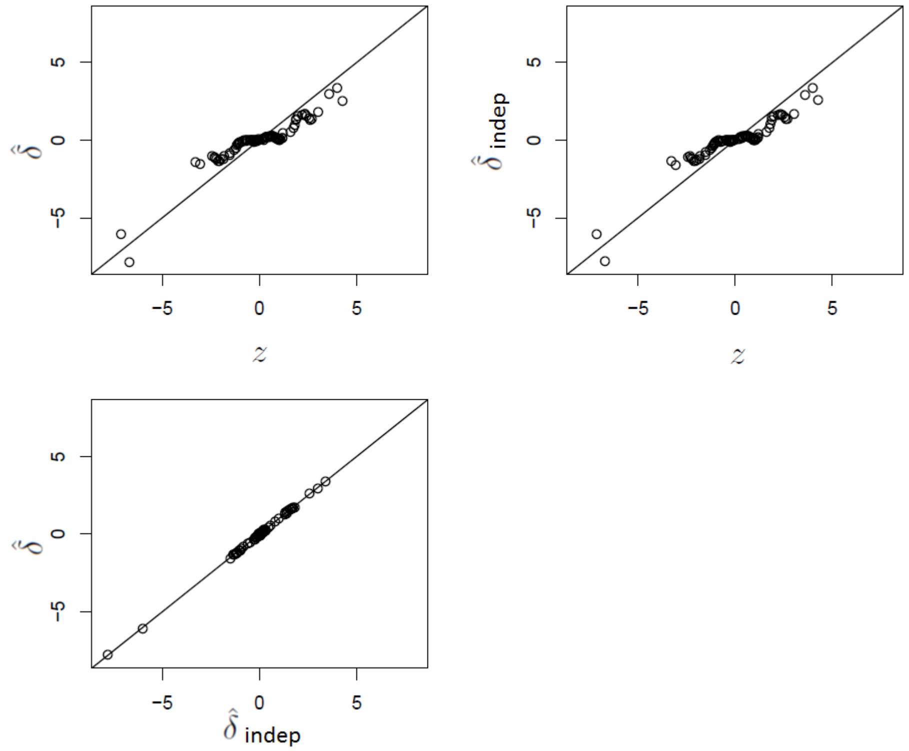

Supplementary Figure 6. *Illustration of the true projack vs. independent projack for* $K = 5$, $m = 100$, $n = 500$

μ values were obtained as sorted values independently drawn from $N(0, 1)$. The figure shows the results of true projack-5 (1000 splits) vs. z (upper left), the independent projack-5 (10,000 simulations) vs. z (upper right), and comparison of the two projack estimates (lower left) show that the results are nearly identical.

Supplementary Figure 6 shows the same scenario, except that here the rows of $\mathbf{C}$ were generated as serially correlated according to an autoregressive AR(1) model, with correlation of 0.8 for successive rows. Even though the correlation is high, the independent projack behaves very similarly to the true projack (see Figure), illustrating that the independent projack has utility even when the independence assumption is violated.

7. Appendix G. Analysis of top SNPs, and another real dataset. When the entire $\mathbf{z}$ vector is available, the independent projack is used directly. If only the significant SNPs in genome-wide association (e.g. those

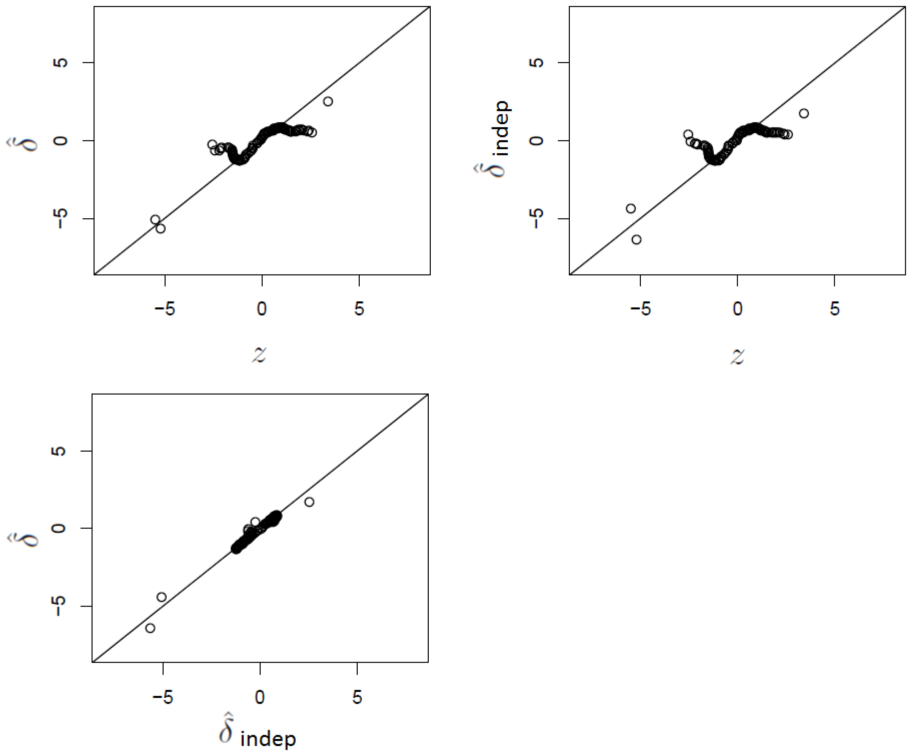

Supplementary Figure 7. *Illustration of the true projack vs. independent projack for* $K = 5$, $m = 100$, $n = 500$, *with serial correlation 0.8 across rows of the matrix* $\mathbf{C}$

with $P < 5 \times 10^{-8}$) are reported, we propose modifying projack by using the significant z-statistics, augmented by an artificial "null" z-vector. For m SNPs, a fixed null vector is given by $z_i = \Phi^{-1}(i/(m_{null} + 1))$, where $m_{null} = m-$(number of significant SNPs), which is then concatenated with the significant z-values and then independent projack used. However, for the psoriasis data reported in the main manuscript and for most reports of significant GWASs, often only the most significant SNP within each genomic region is reported. When only the "top" SNPs are reported in each region, the number of significant SNPs is under-reported, and using m_{null} can cause over-shrinkage. As a practical solution, if a fixed significance threshold α_{thresh} has been used, we use an upper bound $m_{null} = \min(m, 0.05/\alpha_{thresh})$. For the commonly used $\alpha_{thresh} = 5 \times 10^{-8}$ (near the threshold suggested by [1] for arbitrarily dense SNPs) , the upper bound is 1 million, which is justified on the grounds that SNP genome-wide scans correspond approximately to 1 million independent tests.

Here we analyze the data on genetic association of SNP variants associated with hypospadias, reported in [2]. The initial GWAS consisted of 1006 cases and 5846 controls, with a replication sample of 1972 cases and 1812 controls. Twenty-two SNPs, representing the most significant SNPs within each significant region were reported, where $\alpha = 5 \times 10^{-8}$ was used as a significance threshold. Using projack-5, we have the results shown in Supplementary Figure 7 (left panel), also shown in comparison to the naive estimates. The right panel of the figure shows the projack mean squared error performance as a function of K, minimized at $K = 7$. In comparison, the conditional likelihood approach has much larger mean squared error (conditional likelihood result for $P < 5 \times 10^{-8}$ is off the plot and not shown). Again, projack for k in the range 5-10 appears to have the best performance, and considerably better than competing methods.

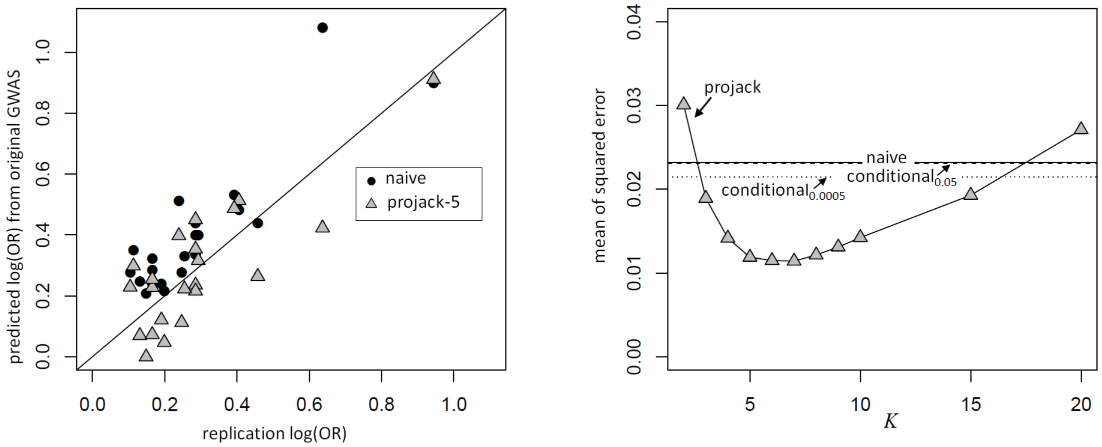

Supplementary Figure 8. *Results from hypospadias GWAS, with replication analysis of 22 significant SNPs. Left panel: log odds ratios in original GWAS and after projack-5 correction, vs. replication log odds ratios. Right panel: Projack-K squared error (estimated values from initial GWAS vs. replication) averaged over the 22 SNPs, vs conditional likelihood and naive approaches.*

References.

[1] Dudbridge, Frank and Gusnanto, Arief. (2008). Estimation of significance thresholds for genomewide association scans. *Genetic epidemiology* **32**(3), 227–234.

[2] Geller, Frank, Feenstra, Bjarke, Carstensen, Lisbeth, Pers, Tune H, van Rooij, Iris ALM, Körberg, Izabella Baranowska, Choudhry, Shweta, Karjalainen, Juha M, Schnack, Tine H, Hollegaard, Mads V *and others*. (2014). Genome-wide association analyses identify variants in developmental genes associated with hypospadias. *Nature genetics*.

[3] Miller, R. G. (1974). The jackknife - a review. *Biometrika* **61(1)**(1-15).

[4] Purcell, S., Neale, B., Todd-Brown, K., Thomas, L., Ferreira, M. A., Bender, J. D. Maller, Sklar, P., de Bakker, P. I., Daly, M. J. and Sham, P. C. (2007). PLINK: a tool set for whole-genome association and population-based linkage analyses. *American Journal of Human Genetics* **81(3)**, 559–75.

[5] Salkind, N.J. (2010). *Encyclopedia of Research Design* . SAGE Publications, Inc.

[6] Thorburn, D. (1977). On the asymptotic normality of the Jacknife. *Scandinavian Journal of Statistics* **4(3)**(113-118).

Broughton Hall
6201 Stinson Drive
Campus Box 7655
Raleigh, NC 27695
E-mail: yihui_zhou@ncsu.edu
fred_wright@ncsu.edu
